# Advancements in MRI-Based Radiomics and Artificial Intelligence for Prostate Cancer: A Comprehensive Review and Future Prospects

**DOI:** 10.3390/cancers15153839

**Published:** 2023-07-28

**Authors:** Ahmad Chaddad, Guina Tan, Xiaojuan Liang, Lama Hassan, Saima Rathore, Christian Desrosiers, Yousef Katib, Tamim Niazi

**Affiliations:** 1 School of Artificial Intelligence, Guilin Universiy of Electronic Technology, Guilin 541004, China; 2 The Laboratory for Imagery, Vision and Artificial Intelligence, École de Technologie Supérieure (ETS), Montreal, QC H3C 1K3, Canada; 3 Eli Lilly and Company, Indianapolis, IN 46285, USA; 4 Department of Radiology, Taibah University, Al Madinah 42361, Saudi Arabia; 5 Lady Davis Institute for Medical Research, McGill University, Montreal, QC H3T 1E2, Canada

**Keywords:** radiomics, prostate cancer, mpMRI, Gleason score

## Abstract

**Simple Summary:**

The integration of artificial intelligence (AI) into radiomic models has become increasingly popular due to advances in computer-aided diagnosis tools. These tools utilize statistical and machine learning methods to evaluate various medical image analysis modalities. In the case of prostate cancer, there are multiple areas in the radiomics pipeline that can be improved. This article explores the latest developments in mpMRI for PCa and examines the radiomic flowchart, as well as the fusion of traditional medical imaging with AI to overcome challenges and limitations in clinical applications. Furthermore, it addresses challenges related to radiomics, radiogenomics, and multi-omics in prostate cancer and suggests the necessary critical steps for clinical validation.

**Abstract:**

The use of multiparametric magnetic resonance imaging (mpMRI) has become a common technique used in guiding biopsy and developing treatment plans for prostate lesions. While this technique is effective, non-invasive methods such as radiomics have gained popularity for extracting imaging features to develop predictive models for clinical tasks. The aim is to minimize invasive processes for improved management of prostate cancer (PCa). This study reviews recent research progress in MRI-based radiomics for PCa, including the radiomics pipeline and potential factors affecting personalized diagnosis. The integration of artificial intelligence (AI) with medical imaging is also discussed, in line with the development trend of radiogenomics and multi-omics. The survey highlights the need for more data from multiple institutions to avoid bias and generalize the predictive model. The AI-based radiomics model is considered a promising clinical tool with good prospects for application.

## 1. Introduction

Prostate cancer (PCa) is a malignant tumor of the male genitourinary system, characterized by epithelial cells. It ranks as the most prevalent malignant tumor in men, the second most common cancer globally, and the fifth leading cause of cancer-related deaths in men. The disease is the primary cancer in 112 nations and is responsible for the majority of cancer deaths in 48 countries [1]. According to the latest statistics, in 2020, there were approximately 1.4 million newly diagnosed PCa cases and 375,000 deaths worldwide [2]. Since PCa develops slowly in its early stages, older men, who are at high risk, may not realize that they are affected. Therefore, timely detection is important to reduce mortality rates. Furthermore, early detection and prompt treatment can significantly reduce PCa-related deaths.

PCa can be examined using several methods, including (1) digital rectal examination (DRE), which is the most straightforward and effective method, and critical for the diagnosis; (2) prostate-specific antigen (PSA) test, including total PSA and free PSA; (3) non-invasive ultrasound examination of the prostate, which can detect early internal nodular changes; (4) Computed Tomography (CT) examination of prostate lesions; (5) magnetic resonance examination of prostate lesions; and (6) prostate biopsy, usually performed with transrectal ultrasound guidance. A biopsy involves taking tissue samples from at least 12 sites, which are then examined for pathological changes [3].

The existing detection methods for PCa have limitations and room for improvement. DRE suffers from variability between reviewers, low reproducibility, sensitivity, and specificity, as well as high false positive rates [4]. PSA is a nonspecific blood marker and lacks sensitivity, leading to false negatives and many unnecessary biopsies [5]. Transrectal ultrasound-guided biopsy is prone to random sampling errors and may cause bleeding or infection, making PCa detection more challenging [3]. Furthermore, combining multiple methods may be more effective than using them individually. In this context, there is a need for more precise, accurate, and non-invasive detection methods to improve PCa diagnosis.

PCa patients are typically classified according to their test results (i.e., PSA, DRE, TRUS, and biopsy), and treatment plans are determined accordingly. In addition, the Gleason score (GS) is a widely used classification method. Unlike other cancer grading systems, the GS does not use the worst morphological grade but instead sums up the primary and secondary morphological grades to determine the overall grade. This method provides better prognostic information for PCa patients and is therefore more appropriate for PCa diagnosis [6]. For example, Gleason grade 1 (rare) shows large glands with consistent rules and dense back-to-back arrangement. Gleason grade 2 presents relatively irregular large glands forming small nodules that are not fused. Gleason grade 3 exhibits small acinic glands with infiltrative growth or small cribriform glands. Gleason grade 4 presents fused glands, large cribriform glands, or renal clear cell carcinoma-like morphology. Gleason grade 5 has no adenoid structure, single cancer cell infiltration, or acne-like appearance with cancer cell necrosis [6].

As GS has certain limitations, the International Society of Urological Pathology (ISUP) has proposed a new grading system based on five grade groups (GG) to address these limitations. The morphological definition of the five GGs is as follows: GG 1 (GS ≤6): cancer composed of a single, discrete, and well-structured gland; GG 2 (GS 3 + 4 = 7): mainly composed of discrete and well-structured glands, with a small part composed of poorly shaped, fused, renal globular, cribriform glands; GG 3 (GS 4 + 3 = 7): mainly composed of poorly shaped, fused, renal small globular, cribriform glands, and a small part composed of suitable discrete glands; GG 4 (GS 4 + 4, GS 3 + 5, GS 5 + 3): composed of poorly shaped, fused, renal globular, cribriform glands or discontinuous glands and glands lacking a small part, or glands lacking a small part of discontinuous and well-formed glands; GG 5 (GS 9, GS 10): no glandular cavity formation or glandular cavity necrosis [7]. Proper classification and grading of patients can help clinicians formulate personalized treatment plans and evaluate the prognosis of patients. While prostate biopsy remains the gold standard for detecting PCa, mpMRI is emerging as a useful method in early screening, especially as early clinical symptoms may not be apparent. mpMRI allows a detailed anatomical evaluation of the prostate, provides a clear description of the regional anatomy and acceptable resolution of soft tissue, and has many MRI scan options that are superior to other imaging methods [8]. Specifically, mpMRI is a non-invasive imaging technique that has several applications in PCa detection, localization, staging, risk classification, and biopsy guidance [9]. However, radiologists interpret mpMRI images to diagnose illnesses, including PCa. However, like any medical imaging, the interpretation of mpMRI images is subjective and can be influenced by the radiologist’s experience and expertise. This subjectivity can potentially lead to errors in interpretation. Therefore, obtaining high-quality images and ensuring proper patient preparation is important for obtaining accurate diagnoses. With an increasing emphasis on identifying and treating high-risk tumors and reducing overtreatment of low-risk tumors, mpMRI plays a critical role in PCa diagnosis [8]. Additionally, mpMRI can be used for quantitative imaging (radiomics) to predict clinical outcomes of PCa [9].

**Figure 1:**
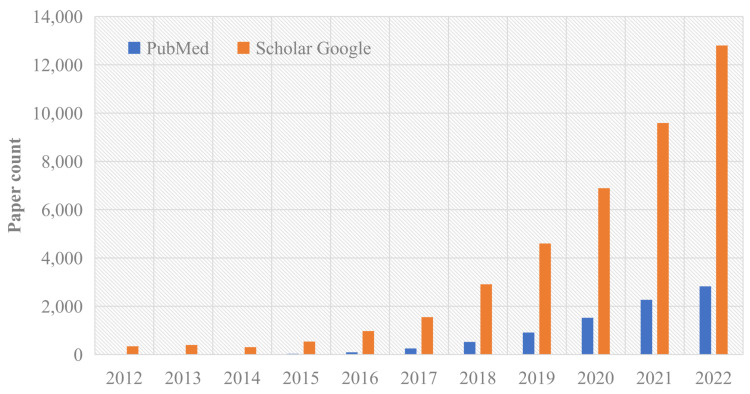
A bar chart demonstrates a steady increase in the number of radiomics publications on PubMed and Scholar Google since 2012. The chart presents the number of radiomic papers on the y-axis and the corresponding years on the x-axis, emphasizing the growth and significance of radiomics research.

Radiomics is a quantitative method used to analyze data obtained from medical images, including mpMRI, to evaluate cancer (e.g., PCa) and other diseases. Radiomics aims to extract a large number of quantitative features from medical images and use these features to establish models that can classify and predict various aspects of cancer, such as diagnosis, prognosis, and response to treatment. Traditional radiology extracts features from a single modality, such as Computed Tomography. However, with the development of artificial intelligence (AI) technology, radiomics is becoming more applicable in the medical field. It can be used to predict the prognosis of multiple cancers, the response to various treatment methods, distinguish benign treatment confounding factors and progression, identify abnormal tumor response, and predict mutations and molecular characteristics. Radiomics is moving towards a multi-parameter approach, enabling tumors to be characterized more quantitatively and objectively to overcome the variability between observers. This may result in helpful predictive biomarkers that cannot be recognized by visual analysis [10]. However, one of the obstacles in translating radiomics from research to clinical practice is the interpretability of the data [11]. Furthermore, the challenges of texture image variability must also be addressed [12,13]. Despite these challenges, radiomics studies have been widely distributed and published, as shown in Figure 1.

In summary, the contributions of this survey can be listed as follows:

- We provide a brief overview of radiomics models used for PCa. A detailed analysis of the key motivations for radiomics applications using current feature extraction, feature selection, and machine learning techniques is also included.
- We commonly analyze the clinical value of mpMRI used in PCa, such as guidance for treatment, showing the pathological areas of tumors, and stating the current challenges with mpMRI.
- We present the development of radiogenomics and multi-omics with PCa applications.
- We discuss the recent challenges related to the current PCa radiomics, radiogenomics, and multi-omics with future directions in these topics.

The remainder of this paper is structured as follows. Section 2 briefly describes the impact of mpMRI for PCa. Section 3 presents the standard radiomic model. Section 4 discusses the stability of radiomics. Section 5 introduces the predictive models for classifying PCa with MRI scans. Section 6 and Section 7 highlight the research value of radiogenomics and multi-omics for analyzing PCa, respectively. Section 8 discusses the future perspective and limitations. Section 9 summarizes the work and contribution of this paper.

## 2. Multi-Parametric MRI Imaging of the Prostate

Multi-parametric MRI represents both anatomical sequences (i.e., T1-weighted (T1W), T2-weighted (T2W)) and functional sequences, including diffusion-weighted imaging (DWI) and dynamic contrast enhancement (DCE). As T1W is limited in evaluating prostate morphology or identifying intraglandular tumors, mpMRI also uses T2W, DWI, and DCE, which have high sensitivity and specificity for detecting significant abnormal tissues. The quality of these sequences depends on the hardware and software used and the scanning parameters chosen, as well as on several other factors, including bowel motility, rectal dilation, the presence of total hip replacement, and post-biopsy bleeding [9].

### Impact of Multi-Parametric MRI

The most important clinical value of mpMRI is to guide the biopsy of the abnormal area of PCa to complete the direct evaluation of the location, size, and cancer stage of different cancers in the prostate. The accuracy of mpMRI-guided biopsy depends on the ability to observe PCa on the mpMRI. A new study compared standard biopsy with MRI-guided biopsy and proved that MRI-guided biopsy has higher sensitivity in detecting PCa with clinical significance, reducing the probability of over-examination and treatment [14]. In addition, men with positive MRI results should also undergo standard biopsy with target biopsy [14,15]. A prostate imaging reporting and data system (PI-RADS) was introduced to collect, interpret, and report standard MRI images. The first version of this system was proposed in 2012, which includes the essential scoring criteria [16]. A second version further refined the system proposed in 2015 [17], and updated PI-RADS v2.1 in 2019, making the system more advanced [18]. Its development promotes the standardization of MRI and contributes to more clinical applications. The scoring system provides a framework for evaluating individual T2W, DWI, and DCE sequences and integrates these individual scores into overall risk assessment categories from 1 to 5. These risk categories contribute to the determination of biopsy [19]. For example, PI-RADS v2.0 scores range from 1 to 5. A biopsy is required for a lesion with a PI-RADS score of 4 or 5. However, it is not required for lesions with a PI-RADS score of 1 or 2. A score of 3 indicates that the lesion may require biopsy, depending on clinical factors [17]. This leads to considering the mpMRI in detecting PCa and the treatment plan. A recent survey has indicated that MRI-based radiomics research on PCa has the potential to enhance the PIRADS report in the future. Specifically, this research may improve the diagnosis and risk stratification of PCa [20].

Table 1 reports recently published literature using mpMRI to detect Pca. According to recent papers presented in Table 1, it can be observed that mpMRI is presently the most frequently used technique for identifying PCa. Specifically, these works showed that using mpMRI can improve the detection rate of clinically significant PCa (csPCa) [14,21,22,23,24]. In [25], they found no significant differences in the detection of PCa and csPCa using MRI in-core and MRI-TRUS fusion target biopsy (TBx). In addition, in terms of MRI imaging, the study of [26] combined MRI with prostate-specific membrane antigen (PSMA) to improve negative predictive value (NPV) and sensitivity of csPCa. In [27], they showed that the use of miniature ultrasound biopsies to detect csPCa was not significantly different from mpMRI. In [28], mpMRI outperforms Foggia Prostate Cancer Risk Calculator (FPC-RC) and is similar to the European Randomized Study of Screening for Prostate Cancer RC (ERSPC-RC) and Prostate Biopsy Collaborative Group RC (PBCG-RC) in predicting csPCa. This leads to improving the diagnostic accuracy of the risk calculator. In addition, the mpMRI risk calculator was studied and proved to avoid unnecessary biopsies [29]. Combined mpMRI with MR spectroscopy improved the performance of PCa diagnosis [30]. A new study showed that the combination of fusion-guided biopsy and systematic biopsy could improve the detection of PCa by 10% and identify csPCa [31]. While in [32], considering MRI-lesion targeted (MRI-TB) in MRI-positive patients improved the detection rate of csPCa.

The role of mpMRI in the guide to treatment includes radical prostatectomy, definitive radiotherapy, and active monitoring [19]. For example, mpMRI images can show the location of the lesion and correctly help in segmenting the tumor volume, which simplifies treatment management [34]. Patients with PCa undergoing surgery or radiotherapy have the possibility of recurrence, including biochemical recurrence (BCR), local recurrence, and distant metastases. Before treatment, we can combine mpMRI scans with clinical variables (clinical stage, PSA, and biopsy Gleason score) to determine the risk of recurrence. Using clinical variables and medical images, the AI model can improve the performance in multitask prediction related to PCa [35,36]. For PCa segmentation, mpMRI images can also improve performance metrics. For example, the combination of T2W and ADC images can enhance the evaluation of PCa in both visual quality and objective assessment [37]. In [38], a deep learning model, “ProGNet” was developed to segment MRI images of prostate tissues automatically. This model, “ProGNet” outperforms U-Net, and radiologic technologists reduce the time to clinically segment the prostate to facilitate targeted biopsy studies while potentially improving biopsy accuracy.

Despite the critical role of mpMRI for PCa management, there are still some challenges. For example, mpMRI is reliable in excluding clinically significant PCa, but whether a biopsy is needed in the case of negative mpMRI is still controversial, especially for young patients [39]. There are significant differences between radiologists when conducting interinstitutional research, which may make the same patient receive different examination results in other institutions [19]. Before mpMRI becomes the standard management for PCa treatment, large datasets derived from multi-centers are required. For patients with mpMRI limitations (i.e., pacemakers, metal implants, and claustrophobia), it is not easy to obtain mpMRI images, as well as some inherent problems of mpMRI, such as variability and challenges in image acquisition and interpretation [40]. All these limitations require more investigation to solve and improve PCa management challenges.

## 3. Radiomics Analysis for PCa

**Figure 2:**
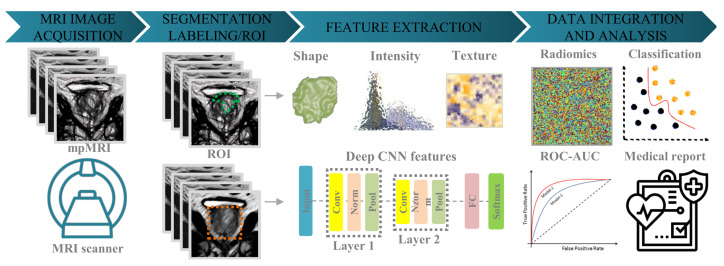
A standard radiomic pipeline typically involves four key steps. First, an MRI scanner captures multi-parametric MRI images. Second, the images are segmented to label abnormal regions or areas, including the region of interest (ROI). Third, texture, shape, and intensity features (and/or deep features are extracted from convolution neural network (CNN) layers)are extracted from the images. Finally, the imaging features are aggregated with relevant clinical variables using a classifier model to predict clinical tasks such as Gleason score of prostate cancer.

By avoiding the need for invasive procedures, such as obtaining pathological specimens through surgery, radiomics offers a more patient-friendly option. For example, radiomics can provide information without causing undue discomfort to patients. To facilitate the use of radiomics, we present the standard steps of the method as illustrated in Figure 2.

### 3.1. Image Acquisition

Radiomics leverages various medical images, such as ultrasound, X-ray, CT, MRI, and PET scans. Numerous public datasets, such as The Cancer Imaging Archive (TCIA), provide detailed manual annotations of medical images, including Gleason scores and recommended treatments, labeled by clinicians (and/or radiologists, oncologists, and pathologists). Among these modalities, MRI is a preferred one due to its superior soft tissue imaging and sensitivity to metastases, making it a popular choice for prostate examinations [41]. However, image acquisition is a critical factor in the radiomic pipeline. Limitations in technology or equipment may result in some biological defects not being displayed, leading to unreliable results. To address this issue, initiatives such as the Quantitative Imaging Biomarkers Alliance (QIBA) [42], the International Biomarker Standardization Initiative (IBSI) [43] and the European Imaging Biomarkers Alliance (EIBALL) [44] have been proposed to promote quantitative imaging and ensure reliability. These initiatives specify measurement accuracy requirements for quantitative imaging biomarkers and outline procedures to achieve optimal accuracy while minimizing possible biases.

The extraction of radiomic features from medical images requires a series of preprocessing steps to enhance the quality of the data. These steps are necessary because the accuracy and reliability of the extracted features heavily depend on the quality of the input data. Denoising is one of the preprocessing techniques that is commonly used to reduce the noise in the data. The presence of noise can negatively impact the accuracy of the radiomic features, which is why denoising is an important step. Another important technique used in data preprocessing is standardization, which involves scaling the data to a common range. This technique is particularly useful when dealing with data from different sources or modalities because it makes the data comparable. Resampling is also a widely used technique that involves adjusting the resolution of the data to a common scale. This technique can improve the accuracy of the features by ensuring that the data are uniform. Several methods have been proposed to achieve data normalization, such as linear variation, Gaussian, and z-values, among others. These techniques have been shown to significantly impact the results of predictive models [45]. After completing the image acquisition and preprocessing steps, the next step in the radiomic process is to label the region of interest, such as lesion regions. This step is accomplished using segmentation techniques, which identify and separate the region of interest from the surrounding tissues. Specifically, the combination of these preprocessing techniques and segmentation provides a solid foundation for the accurate and reliable extraction of radiomic features.

### 3.2. Segmentation

Segmentation of PCa in MRI images is the process of identifying and isolating cancerous tissue within the prostate gland using MRI scans. Specifically, MRI scans of the prostate provide high-resolution images that can be used to distinguish between cancerous and non-cancerous tissue. However, interpreting these images can be challenging due to the complex anatomy of the prostate gland and the variability of cancerous lesions. Segmentation techniques aim to automate this process and improve the accuracy and efficiency of diagnosis and treatment. Several approaches have been developed for PCa segmentation in MRI images, including manual segmentation by radiologists, semi-automated methods using thresholding and region growing, and fully automated methods using machine learning algorithms [36,46]. Machine learning algorithms such as convolutional neural networks (CNNs) have shown promising results in segmenting PCa in MRI images with high accuracy. The process of segmentation involves the identification and separation of regions of interest (ROI) in both two-dimensional (2D) and three-dimensional (3D) space, also referred to as the volume of interest (VOI). Accurate ROI labeling is an important step in studies where pathological regions require precise boundaries, which can be challenging during the segmentation. However, automatic segmentation algorithms for ROIs require improvement. Manual segmentation is time-consuming and depends on the size of the data set. Both manual and semi-automatic segmentation can be affected by observers, leading to deviations. Therefore, the reproducibility of radiomics features derived from manual or semi-automatic image segmentation and correction should be evaluated for intraobserver and interobserver variability, and non-reproducible elements should be excluded from further analysis [47]. Fully automatic segmentation is expected to become the dominant method soon [48]. For example, CNNs have been widely employed for automatic segmentation [49,50]. In [51], they developed a multiregional automatic segmentation model based on CNNs using the intercontinental queue of PCa MRI. In [52], end-to-end CNNs were proposed to automatically segment csPCa lesions, and the accuracy of the segmentation results was higher than other methods (Dice and sensitivity were 0.7014 and 0.8652, respectively).

Additionally, CNN (V-Net T2) and Active Shape Model (ASM) increase the Dice Similarity Coefficient (DSC) value from 0.840 to 0.851 and reduce the Hausdorff distance from 10.74 to 7.55 mm, improving the segmentation performance [53]. Despite many advanced contributions in automatic segmentation methods, the semi-automatic segmentation that gives options to clinicians is the most recommended. For example, 3D-Slicer (www.Slicer.org (accessed on 7 April 2023)) and ITKSNAP (http://www.itksnap.org (accessed on 7 April 2023)) tools are used to label tumors.

So far, one challenge in PCa segmentation is the presence of false positives and false negatives, which can lead to over or underestimation of the extent of the PCa. To address this, more work is needed to integrate multiple MRI sequences with advanced image processing techniques to improve segmentation accuracy.

### 3.3. Feature Extraction, Selection, and Construction

After the ROI segmentation is the feature extraction step, which is the core part of radiomics. These extracted features describe biological information and important characteristics of abnormal tissue and are used as input into predictive models. Currently, radiomic features include morphological/shape, texture (such as gray-level co-occurrence matrix (GLCM), gray-level size zone matrix (GLSZM), gray-level run length matrix (GLRLM), gray-level dependence matrix (GLDM), neighboring gray-tone difference matrix (NGTDM), etc.), and high-order statistical features [54]. Joint Intensity Matrix (JIM) [55] and deep texture [56] are proposed radiomic features to predict the Gleason Score (GS) of prostate cancer using mpMRI scans. Shape features in conventional radiomics are typically derived from ROI that have been manually labeled. However, it is important to consider inter-observer variability during segmentation, as this can impact the reliability of selected features. To address this, segmented images can be analyzed by multiple observers and features can be compared using metrics such as intraclass correlation coefficient (ICC) and consistency correlation coefficient (CCC). Only variables that meet specific thresholds for robustness should be selected [57]. With CNN models, features are extracted and selected based on CNN layers (such as feature maps and pooling layers) [3]. The extraction of radiomics features typically results in a high-dimensional feature space. This can lead to overfitting when using the features as inputs to predictive models. The high-dimensional feature space includes redundant and noisy information, which can introduce errors in practical applications and affect accuracy. When the dimension of the feature exceeds a specific limit, the classifier’s performance may decline, and training time will increase. Feature dimensionality reduction is therefore required to reduce errors, improve the efficiency of radiomics feature data, enhance the model’s prediction ability, and shorten training time.

Table 2 reports the recent literature on feature selection techniques. The feature selection methods include Random Forest (RF), the least absolute shrinkage and selection operator (LASSO), principal component analysis (PCA), maximal relevance and minimal redundancy (mRMR), etc. LASSO, PCA, and RF are frequently used methods for feature selection. According to a comprehensive study by Zebari et al. [58], PCA is the most commonly employed algorithm for dimensionality reduction. The feature selection methods can also be divided into the following categories: (1) Filtering method: evaluate the features according to the divergence or correlation of the features, set the threshold, and then select the features, such as correlation analysis, analysis of variance and rank sum test; (2) Wrapper method: select or exclude several features according to the objective function, such as the recursive feature elimination method; and (3) Embedding method: firstly, the algorithm and model of machine learning are used for training to obtain the weights of each feature, and then the features are selected according to the weights, such as logistic regression [59]. Despite the feature selection methods’ advantages, more investigation is still needed to solve the overfitting problem.

### 3.4. Building Predictive Models

Modeling methods in radiomics can be divided into unsupervised and supervised categories. Unsupervised methods, such as k-means clustering and hierarchical clustering, are used for datasets without labels, while supervised methods, such as random forest, support vector machine, artificial neural network, and logistic regression, are used for labeled datasets. While no single classification method has been identified as universally superior in radiomics, supervised methods are generally used more frequently. For example, the logistic regression model is often preferred for its simplicity and has become the most commonly used method for building models.

The standard practice is to split datasets into training (70%) and test (30%) groups. The model is constructed using the training dataset and is fine-tuned through internal validation methods like k-fold cross-validation. The test datasets are used to evaluate the performance of the predictive model [73]. A variety of metrics can be used to assess the model’s performance, including the area under the receiver operating characteristic (AUC-ROC), sensitivity (SE), specificity (SP), accuracy (ACC), and decision curve.

Many modeling techniques commonly used in radiomics are reported in Table 3. In [74], a radiomic model based on quantitative imaging features is used to predict clinically significant PCa. In [75], the radiomics model with mpMRI has been proven to help improve the diagnostic performance of PI-RADS v2.1 in PCa. In [61,76], radiomics is used to actively monitor the progression of PCA. In [77,78], the radiomic model was used to study the risk of lymph node invasion in PCA patients to avoid expanding pelvic lymph node dissection. In addition, radiomic models based on combination PET + ADC scans have complementary values [79]. In [80], the 3T-DWI b2000 sequence was used for the prognosis and targeted biopsy, proving ADC’s feasibility for PCa detection. In [60], the nomogram shows the radiomic model with MRI and PI-RADS as a noninvasive method capable of predicting PCa. As reported in Table 3, the radiomics model was used and demonstrated a noticeable improvement in the detection rate of PCa.

## 4. Radiomics Stability

A big challenge facing radiomics is related to model stability [81]. Many factors that may consider in studying stability are (1) feature importance, (2) generalizability, (3) stability testing, and (4) failure examination. For example, features in a radiomic model could be estimated and evaluated regarding the relative importance in the trained model [82]. In addition, testing the model using different patient groups can assess generalizability. Furthermore, the stability and reproducibility of features may be applied through the use of standardized protocol guidelines and software [83]. More details about the radiomic stability are explained as follows.

### 4.1. Feature Importance

To improve the development of a stable model, it is important to identify significant radiomic features that are relevant to clinical tasks. This may involve identifying and avoiding redundant features that can lead to scalability issues. By excluding highly correlated or redundant features, a more stable model can be built [84,85]. To minimize the risk of using unstable and unrepeatable features in the radiomics analysis, it is suggested to retest the analysis of the treatment site, scanner, and imaging protocol control to evaluate and analyze the impact of each factor on the model [86]. In addition, the radiomic features also require being stable when various data sources are used.

### 4.2. Generalizability

Multicenter, large sample data, and additional clinical features are required to achieve the model’s generalizability. For example, it may be beneficial to train the model with diverse groups of patients derived from multiple sites. This approach can help to increase the model’s ability to perform well on unseen data and in different clinical settings. Training the model on a single group of patients from a particular site may lead to overfitting, which can limit the model’s performance on new data. However, training the model with patients from various sites can help capture the heterogeneity in the radiomic features across different patient populations and imaging protocols. Since data may come from different imaging acquisition protocols or devices in clinical applications. Radiomic models with other patients should consider these factors when training models [87]. In [88], they evaluated the generalizability of the model using two external datasets and found a significant decrease in performance compared to internal cross-validation (average AUC 0.54 vs. 0.75). In [89], federated learning can improve the generalization performance of PCa models across institutions and protect data privacy. So far, domain adaptation and federated learning can be valuable techniques for enhancing the generalizability of machine learning models, particularly in the context of medical imaging, where data can be diverse and challenging to obtain [90].

### 4.3. Stability Testing

As previously mentioned, stability can be assessed through reproducibility. For mean or median comparison, common indicators were used, including CCC, coefficient of variation (CoV or CV), Pearson or Spearman correlation, and parametric or nonparametric statistical tests (*t*-test, analysis of variance test, Wilcoxon test, Friedmann test, etc.) [91]. Many studies performed radiology-assisted experiments to improve repeatability and reproducibility and identify stable features. However, unstable features may also contain relevant information needed for research, leading to an overestimation of model performance. In [92], a data analysis method was proposed to evaluate the stability of the radiomics features obtained from MRI. This method shows that a large part of the radiologic features based on ADC (25–29%) show retest stability in various tissues, MR systems, and suppliers. In addition, different observers and even the same observer may have varying evaluations of the same image, which can result in variations in the results. For instance, in a study where a pathology team of four experts evaluated 425 internal biopsy tissues [93], two European pathologists exhibited an observer consistency of 0.89 quadratic-weighted kappa ($K_{quad}$), while the consensus among general surgical pathologists was 0.69 $K_{quad}$. The consistency between uropathologists and general surgical pathologists ranged from 0.50 to 0.59 $K_{quad}$. To reduce errors, it is possible to train observers, standardize techniques and judgment criteria, estimate the degree of non-compliance between observations, and randomly assign patients to observers.

### 4.4. Failure Examination

Failure examination can show us the potential defects in established radiomics models. A summary of the relevant radiomics stability studies is presented in Table 4. It requires establishing a quality management procedure to check whether the model is still valid after updating [87]. In [94], 2D-based radiomic features of MRI models showed good stability in identifying GS. In addition, image normalization may be considered a stability factor in data preprocessing. For example, normalization is also applied in multisource data for prognosis modeling [95]. Multi-modal radiomics models have been developed and customized according to specific radiology protocols, which can solve particular problems related to single and multi-center research [96]. In addition, the radiomic features of phantoms and volunteers with low COV and high ICC can be considered good candidates for MRI radiomics studies [97]. With the progress of stability radiomic techniques, more investigation to manage these techniques with minimization of model bias is recommended.

## 5. Radiomics Related to Prostate Cancer

Many machine learning algorithms have been used for classifying prostate lesions using MRI images. For example, the PCa classifications may be related to malignant versus (vs.) benign, csPCa vs. clinically insignificant prostate cancer (ciPCa), multi-class of invasiveness (aggressive, indolent, and indeterminate), GS groups, etc. In [102], they combined texture features derived from T2W images and ADC maps using a support vector machine to classify between low and high aggressive cases of PCa, which showed a higher AUC value with 0.96 compared to the use-only ADC map with 0.55. In [102], a fully automatic computer-aided diagnosis system has been developed, which can correctly identify patients with invasive PCa, and it can eliminate the need for manual segmentation and analyze data sets from multiple centers. In [103], they presented an algorithm model that combines radiomics and pathology to differentiate between indolent and aggressive cancers on MRI-CorrSigNIA, which achieved an accuracy of 80%. Another study aimed to predict GS and established a radiomics model using T2WI, ADC, and diffusion kurtosis imaging (DKI) sequences. The radiomic model using imaging features with lesion size and PI-RADS score predicted PCa with GS≥8 [70]. Using MRI images, the radiomics model could distinguish between csPCa and ciPCa [71]. In addition, the radiomics model using DCE-MRI sequences with logistic regression in predicting the aggressiveness of PCa showed a feasible diagnostic performance [72]. Compared with T2WI and DWI sequences, prostate DCE-MRI could better display the tumor boundary, which is beneficial to the segmentation of the ROI. However, the study only focused on the radiomics features of the DCE-MRI sequence, and future studies need to be combined with other sequences to improve the diagnostic performance of the radiomics model [72]. Chaddad et al. proposed a new radiomic signature based on the joint intensity matrix (JIM) to predict the Gleason score (GS) of prostate cancer (PCa) patients. The predictive model achieves an AUC value of 78.40% for GS≤6, 82.35% for GS=3+4, and 64.76% for GS≥4+3 [55]. In another study, texture features used with a random forest model achieved an average of AUC of 83.40%, 72.71%, and 77.35% to predict GS=6;6<GS<3+4 and GS≥4+3, respectively [54]. The performance metric in predicting the GS is significantly improved when the imaging features are extracted from CNN layers, known as deep radiomic features [56]. With the related approaches to PCa, more investigation is still needed to consider all MRI sequences with AI models in monitoring patients with PCa. To reduce the gap between the academic research of AI in PCa and the improvement of the interpretability of AI models in clinical diagnosis support. It is suggested to solve the limited labeled data, complete the further development and validation of multi-reader research and prospective evaluation, and formulate and improve the standard evaluation criteria [104].

## 6. Radiogenomics in Prostate Cancer

The improvement of gene expression levels has strongly promoted the rapid development of genomics. By combining imaging and genomics data, radiogenomics provides a more accurate method for diagnosing and avoiding overtreatment of low-risk tumors [56]. Specifically, radiogenomics may use imaging features to predict (or combine) the status of genes and guide the diagnosis, treatment, and prognostic process of PCa [105]. For example, the combination of mpMRI and gene expression data can detect the radioactive signature of PCa. Because of the susceptibility of gene mutations in PCa, many genes are included in gene testing guidelines to assess the risk of PCa and provide guidance for targeted personalized therapy. Common genes used as biomarkers include the breast cancer (BRCA) gene, E-twenty six(ETS)-related gene (ERG), hypoxia gene, ATM gene, etc. Identification of BRCA mutations can be used for PCa screening strategies, in which BRCA 1 and BRCA 2 are key genes associated with PCa susceptibility and are related to hereditary breast cancer and ovarian cancer syndrome [106]. ERG is the result of a fusion of the androgen receptor-regulated transmembrane protease serine 2 (TMPRSS2) with proto-oncogenes. Hypoxia is an essential feature of the tumor microenvironment, which affects the treatment and prognosis of PCa. Hypoxic gene signatures are usually based on gene expression responses in cell lines exposed to hypoxia. In [107], the risk marker constructed by two hypoxia and immune-related genes, ISG15 and ZFP36, showed significant PCa prediction ability and was helpful to the prognosis of PCa.

Table 5 presents recent radiogenomic studies of PCa and their findings. In [108], PTEN and ERG were found to be correlated with PCa visibility on MRI. In clinical trials, prophylactic PCa resection is the primary prevention choice for BRCA 2 carriers [109]. Detecting BRCA gene mutations in PCa patients helps guide treatment and further genetic detection [110]. HP${}^{13}C$-MRI can distinguish inactive from aggressive PCa based on unique metabolic features [111]. The visibility of mpMRI increased when the tumor evolution resulted in numerous protein groups different from normal PCa [112]. Ragnum-signature has been further developed as a biopsy-derived hypoxia biomarker for PCa [113]. The combination of sSelectMDx and PI-RADS is more sensitive in detecting PCa and may avoid unnecessary biopsy [114]. Early gene mutation detection, including BRCA 1/2, can improve the survival rate of patients [115]. Furthermore, the RNA sequencing of benign biopsies revealed the upregulation of NKX3-1 and HOXB13 in the absence of T cells, which may help identify a higher risk of PCa [116]. Hyperlipidemia is associated with invasive features of PCa without TMPRSS2-ERG fusion or PTEN deletion/mutation [117]. Eleven miRNAs were identified as sensitive biomarkers for early detection of clinically significant PCa [118]. Additionally, recent research has found that ANGPTL4, VEGFA, and P4HA1 (hypoxia-related genes) are related to PCa texture features [119]. In [120], Fischer et al. identified four biomarkers belonging to genes and miRNAs that play important roles in PCa, which have the ability to differentiate between T2c and T3b stages. Benafif et al. [121] demonstrated the feasibility of using germline SNPs in targeted PCa population screening in the UK community through the BARCODE1 study. So far, these studies demonstrate the importance of radiogenomic research in understanding PCa and identifying potential biomarkers for early detection, risk assessment, and treatment guidance.

Furthermore, genomic measurements are typically assessed on a small tumor. They reflect only one aspect of tumor heterogeneity. With the ability to determine tumor heterogeneity, radiogenomics offers a personalized approach to risk stratification in patients with PCa [122]. It can also guide clinical treatment strategies based on individual clinical risk factors. For example, one of the personalized methods of PCa risk calculation is to include clinical data of patients, consisting of PSA levels, and PCa Antigen 3 (PCa3) and TMPRSS2-ERG (T2:ERG) expression [105]. Due to the limited medical datasets, the short-term solution is to use transfer learning or data augmentation, and the long-term solution is to use multi-institutional data by facilitating the development of online databases [123]. Personalized treatment requires sequencing a patient’s genome, transcriptome, or proteome [124]. Using genome sequencing to classify cancers and identify tumor patients with actionable goals may help clinicians make more accurate treatment decisions. Targeted sequencing is currently used to detect genetic changes. The development of next-generation sequencing (NGS) technology is a major advance in a different aspect. It will help in recording unique genetic alterations, enabling the generation of large datasets of genomic, transcriptomics, and/or epigenetic features of tumor cells. As known, DNA or RNA sequencing can help detect changes in gene expression features and gene mutations in cancer. RNA sequencing can help identify and produce new long non-coding RNA and gene fusion in PCa. DNA sequencing becomes more sensitive and scalable with the help of NGS. Genome-wide association studies (GWAS) generate large amounts of genomic data and link these data to related cancers like PCa. Thus, integrating data from genomes and radiomics helps to understand their correlation. In [125], a web-based platform ImaGene analyzes the correlation between oncology and imaging data sets by inputting them and building an AI model. Although radiogenomics improves model performance by combining genomic and imaging data, data heterogeneity mainly coming from data source inconsistencies between radioactivity and genomes may be considered a challenge.

## 7. Multi-Omics for PCa

Omics is the comprehensive and quantitative analysis of molecular classes in biological samples. It includes genomics, epigenomics, transcriptomics, proteomics, and metabolomics analyses. Omics is the holistic study of a medical problem from a biological point of view to better achieve a predetermined clinical effect through a single model or a specific feature. It can be used to understand and define changes in biomolecules as complex diseases develop and change. Scientists can search for associations between organisms by analyzing these complex biological macromolecules and constructing accurate disease biomarkers. Multi-omics is to combine these different types of omics data to determine the universal disease–pheno–envirotype relationship or association. Gene expression signatures are the gold standard to guide clinical decision-making, but some questions remain about their clinical utility and interpretability. In 2003, the human genome project was completed, and the information contained in the DNA sequence was deciphered [126]. Thus, omics data associated with the genome, transcriptome, proteome, epigenome, and metabolome rapidly increased. Furthermore, as the technology matures and costs decrease, the likelihood of using omics data to guide clinical practice increases.

We note that epigenomics studies genome modifications, which affect gene expression without altering the DNA sequence. Epigenetic regulatory mechanisms controlling gene expression in PCa mainly include DNA methylation and histone post-translational modifications. DNA methylation is predominantly seen at GPG dinucleotides and leads to gene silencing [127]. Histone post-translational modifications can enhance or attenuate gene expression [128]. These studies facilitate the discovery of new biomarkers or new targeted drugs. In contrast, transcriptomics aims to study the situation of gene expression at the RNA level. Gene signatures of the PCa cell lines LNCaP and VCaP with pre-existing or treatment-induced resistance have been established using single-cell sequencing [129]. For example, a single-cell transcriptomic study identified a population of luminal cells with progenitor functions as a possible contributor to prostate carcinogenesis [130].

In addition, proteomics essentially refers to a protein at a large-scale level, including the expression level of the protein, post-translational modifications, and protein–protein interactions. It provides knowledge about disease occurrence that is gained at the protein level. Proteomics also can discover new molecular biomarkers, which have high clinical potential, especially for routine monitoring because their expression can reflect disease activity in real-time [131].

Metabolomics is a way to quantify metabolites in an organism and find a relationship with physiopathological changes. Analytical techniques are mainly based on nuclear magnetic resonance spectroscopy and mass spectrometry. For example, metabolomics has led to a renewed focus on urine as a valuable biomarker because PCa cells or their substances can be found in prostate fluid. This leads to detecting PCa in urine samples [132]. Moreover, metabolomics studies can lead to a better understanding of disease pathogenesis and therefore better interventions [133]. For example, 26 metabolites were significantly altered in PCa tissues, indicating dysregulation of 13 metabolic pathways associated with PCa development. The most affected metabolic pathways were amino acid metabolism, nicotinate, nicotinamide metabolism, purine metabolism, and glycerophospholipid metabolism [134]. In contrast, the multi-omics study can better describe cancer progress [135], help us to have a more comprehensive view of factors leading to pathological changes [136], develop new biomarkers, and improve clinical management of patients [137,138]. Despite advances in multi-omics analysis, radiomic with multi-omic topics is still limited. More investigation in this direction will detect more biomarkers of PCa.

Table 6 lists the recent literature on multi-omics in PCa, including the specific type of omics, research objectives, and experimental results. As reported, we observe that the results of multi-omics studies are superior to the single omics, multi-omics are very extensive, and the specific methods are also quite different. For example, multimodal molecular analysis based on cell network biology provides robust prognostic biomarkers to detect and identify high-level diseases [139]. While in other studies, multi-omics analysis, integrating genomics, methylomics, and transcriptomics are used to assess the risk correlation between DNA methylation and PCa [140]. Therefore, we suggest explaining the incidence and prognosis of PCA from multi-omics dimensions.

## 8. Future Perspective and Limitations

Radiomics with PCa is gaining increasing attention as a research direction. While MRI is the primary modality used in current radiomics studies of PCa due to its broad clinical application, there remain numerous challenges in future research and application.

Firstly, the majority of current radiomics studies on PCa are single-center, retrospective studies with small sample sizes, which can limit the accuracy of the research results. Therefore, there is a need for multi-center, prospective studies with larger sample sizes to further validate the research findings.

Secondly, DCE-MRI sequences are commonly included in clinical prostate MRI scans, but most current radiomics studies of PCa do not incorporate these sequences. It is suggested to include DCE-MRI sequences to improve the efficiency of image data utilization.

Thirdly, while manual segmentation is currently the primary method used for delineating the region of interest, automatic segmentation algorithms for the prostate could be improved. This is significant in the clinical practice of oncology, as automatic segmentation can enhance the accuracy of biopsy positioning and allow for more precise and repeatable evaluation of metastatic lesions [148].

Finally, since most prostate lesions have low malignancy, prostatectomy is not typically performed, and the diagnosis of suspicious lesions relies heavily on pathological examination. However, inaccurate pathological results from a needle biopsy can negatively impact the diagnostic performance of radiomics models, which rely on pathological findings.

Early detection of most cases of PCa is highly challenging. Currently, the primary means of diagnosing suspected PCa is through pathological examination. However, this process relies heavily on needle biopsy, which carries a risk of missed or incorrect diagnoses, leading to inaccurate results. These errors in pathology directly impact the diagnostic accuracy of radiomics. Therefore, enhancing the precision of pathological examination can help improve the performance of radiomics models.

As is widely acknowledged, the approach to treating tumors depends on a range of factors, including the tumor’s pathological type, disease stage, patient condition, cytogenetic changes, and other considerations. The efficacy of treatment can vary from patient to patient, and before administering genomic targeted therapy, a gene test is typically required. Additional assessments, such as radiomics, may also be necessary during treatment to monitor the development of drug resistance. It is noted that gene testing remains a costly, invasive, and time-consuming procedure [149], whereas radiomics is a relatively inexpensive and non-invasive alternative. As a result, genomic targeting may not be a viable treatment option for all patients with PCa.

Radiogenomics associates imaging data with genome maps, but the availability of these data is affected by the databases (e.g., TCGA, TCIA) and the heterogeneity of tumors. It also requires standardization of imaging and biochemical techniques for analysis to identify stable and repeatable biomarkers. Obtaining reliable results requires many queues and biological sample collection to ensure stability.

Advances in omics, such as genomics, transcriptomics, proteomics, and metabolomics, have begun to enable personalized medicine at the highly detailed molecular level. However, omics alone cannot capture the entire biological complexity of most human diseases. Integrating multiple omics features (radiomics + radiogenomics + omics) provides a more comprehensive view of biology and disease [135]. In addition, few studies performed biomarker validation, but few used independent sample cohorts to exclude false positives caused by sample collection and processing. Moreover, the discovery cohorts were small due to the need for standardized methods for sample collection and processing, data acquisition, and bioinformatics analysis. Finally, few studies shared the same biomarker candidates [131]. These challenges require a massive investigation and a collaborative way to share the findings between federated hospital systems.

AI techniques rely increasingly on large datasets, especially when the data are suitable. It is important to note that data sets have varying feature distributions, and differences arise across various techniques when multiple data sources are used. Therefore, the process of identifying and preprocessing appropriate data can result in more valuable research outcomes. Table 7 lists the most common and public data sets that can be used for PCa studies, such as MRI images containing benign and malignant labels acquired by different types of scanners, consisting of manual labels, distinguishing csPCa from ciPCa, clinical variables, examination, diagnosis, and treatment including PSA and other biochemical data, microscopic scans of prostate biopsy samples with imperfect labels and large images.

In the past, open-source datasets were typically constructed to meet specific research needs, which may not align with current research requirements. To better serve a wider range of communities, it is preferable to provide clean data in multiple formats. However, current datasets face several challenges such as low data reading rates, the presence of multiple data types, and complex data processing requirements. In the near future, researchers are likely to adopt a responsible approach to data collection and annotation, as well as data set maintenance and problem formulation, in order to mitigate these challenges [162].

The translation of radiomics models constructed from medical images into clinical applications faces challenges in terms of interpretability. Specifically, there is a lack of transparent explanation regarding the relationship between selected features and clinical outcomes. In order to ensure interpretability and assist clinicians in making clinical decisions, it is important to have a thorough understanding of the decision-making process behind the radiomics process, especially before incorporating AI fields like DL methods. Without adequate interpretability, the decision-making process and possible biases are not well accounted for, leading to the limited clinical utility of radiomics features and models. The General Data Protection Regulation (GDPR) law in the European Union requires an explanation of an algorithm’s decision-making process, and data subjects are entitled to meaningful information about the logic involved [163]. Explainable Artificial Intelligence (XAI) can help to interpret the information behind the “black box” model, showing how the decision was made transparently, thus enhancing the credibility of the model. Different XAI techniques, such as class activation map (CAM), local interpretable model-agnostic explanation (LIME), Shapley additive explanations (SHAP), Gradient-weighted class activation mapping (Grad-CAM), Attention, and Saliency, can be used to improve algorithm performance [90,164]. The explanation forms generated by XAI can be categorized as feature-based, text-based, and example-based, and improve the credibility of AI from different levels. Interpretable methods have been associated with various tasks in radiomics, including image segmentation, lesion and organ detection, image registration, computer-aided diagnosis and staging, prognosis, radiotherapy planning, disease progression monitoring, classification, and image reconstruction [165]. In one study, multi-modal volumetric concept activation was used to provide an explanation, which showed that the detection was mainly based on the location of metastatic PCa in CT anatomy, and the reliability of PET detection was high [166]. In another study, a model fused with multiple DL methods was used to examine PCa with MRI images, and then XAI explained how the model differentiated benign or malignant PCa [167]. We note that many other radiomics, AI, and radiogenomics works could be also discussed. However, this study collects the most common models that are used for PCa analysis.

Radiomics analysis based on mpMRI can not only improve the detection rate of PCa but also predict prognosis, its texture features can reflect the heterogeneity of lesions. After radiation therapy, radiomics during the follow-up process can be used to evaluate the efficacy of treatment and tumor recurrence. When comparing the radiomic results before and after treatment, tumor shrinkage, tissue recovery, and the presence of residual or new lesions can be evaluated, helping determine whether further treatment is needed, thus improving the survival rate. The combination of mpMRI and Prostate Health Index (PHI) in radiomics may help to better estimate the risk categories of prostate cancer at the initial diagnosis, thus achieving personalized treatment methods [168]. We note that the prediction of cancer prognosis is based on statistical data and models, which provide a probability estimate rather than an absolute prediction. Everyone’s cancer situation is unique, including pathological features, health status, and personal factors, all of which may have an impact on prognosis. Therefore, predicting cancer prognosis should serve as a reference for auxiliary decision-making, rather than the only basis. The final treatment decision should comprehensively consider multiple factors and make personalized choices based on individual circumstances.

As PCa is increasingly being diagnosed at an early stage, with excellent survival rates, the rationale for patients’ primary treatment selection has switched to health-related quality of life (HRQOL). Use mpMRI to detect suspicious PCa before biopsy, thus reducing the number of unnecessary biopsies and avoiding the risk of overdiagnosis and overtreatment. At the same time, through the combined method of systematic and fusion targeted biopsy, the detection rate of PCa can be further improved and the risk of missing csPCa can be reduced [169]. Studies usually conduct follow-ups or send questionnaires (e.g., the Expanded Prostate Cancer Index Composite (EPIC) questionnaire and the Short-Form 12 Item Health (SF-12)) at baseline 3, 6, 12, and 24 months after treatment to collect patient-reported QOL outcomes. The EPIC complements existing instruments by measuring a broad range of urinary, bowel, sexual and hormonal symptoms, allowing for a more comprehensive assessment of important HRQOL issues in contemporary PCa management [170]. In [171], they examined a prospective serial cohort of low-dose-rate (LDR) brachytherapy for PCa using MRI and explored factors associated with toxicity and QOL, as assessed by EPIC and the International Prostate Symptom Score (IPSS). In [172], a prospective phase II clinical study was developed to evaluate outcomes in patients treated with MRI-guided whole-gland prostate high-dose-rate brachytherapy (HDR-BT) augmentation with an assessment of toxicity and HRQOL outcomes. In [173], the HRQOL of early PCa patients who did not receive hormone therapy within 3 years after radiotherapy was examined using the 15D instrument and the FACT-P questionnaire, and the HRQOL was the same in the radiotherapy group and the age-standardized general male population. The treatment of PCa is mainly for curative purposes, but the treatment options are usually accompanied by high morbidity of urinary problems and/or erectile dysfunction, significant loss of quality of life, and high treatment costs. Curing PCa, solving possible complications during treatment, and improving quality of life are the common pursuits of doctors and PCa patients. In [174], palliative transurethral resection of the prostate (pTURP) combined with intermittent androgen deprivation therapy (ADT) can be used in the treatment of elderly patients with localized PCa to resolve dysuria and improve QOL. The personalized treatment of PCa remains one of the challenging areas that require further investigation.

## 9. Conclusions

This paper presents advances in MRI-based PCa radiomics and discusses the steps and details of the radiomic flow chart. It describes the integration of AI with traditional medical imaging for radiomics to address the limitations and challenges of clinical applications, in line with the development trend of the significant data era. Currently, the application of radiomics in PCa extends to almost every patient, from diagnosis to grading of PCa, from adjuvant treatment of PCa to prediction of prognosis of prostate patients. Radiomics, combined with ML methods, could relatively objectively diagnose PCa and predict the treatment effect of patients, which is in line with the concept of precision medicine and personalized treatment. Currently, related studies combine PCa radiomics, and genomics to form radiogenomics. Imaging genomics is expected to become a valuable method for detecting PCa genotypes and will become a tool to assist in the diagnosis and treatment of PCa. With the further development of AI and the improvement of radiomics technology, radiomics will play a better and better role in more fields of PCa, with good application prospects. Future research must improve the versatility and quality of radiomics models with more significant multi-institutional data to complete the promotion and transformation of clinical applications.

## Figures and Tables

**Table 1:** Summary of multi-parametric MRI in detecting PCa.

	Biopsy	Method	Conclusion
[14]	No	MRI-TBx	When detecting csPCa, MRI provides a higher DR than the standard biopsy.
[21]	Yes	Multiparametric ultrasound, mpMRI	Multiparametric ultrasound detected fewer csPCa than mpMRI.
[22]	Yes	MRI-TBx, SB	When detecting csPCa, MRI-TBx provides a higher DR than SB.
[23]	No	MRI-TBx, TRUS-Bx	MRI-TBx significantly improved PCa and csPCa DRs more than TRUS-Bx with low or high PSA.
[24]	No	MRI-GB, TRUS-GB	MRI-GB outperformed TRUS-GB in detecting csPCa.
[25]	No	MRI-TRUS fusion, MRI In-core TBx	When detecting PCa and csPCa, MRI In-bore TBx has a higher DR than MRI-TRUS TBx.
[26]	No	MRI, PSMA + MRI	The PSMA + MRI provides higher performance metrics than MRI alone.
[27]	Yes	mpMRI-targeted, micro-US-targeted, non-targeted biopsy	The DRs of mpMRI-targeted biopsy is higher than micro-US-targeted and non-targeted biopsy (completion sampling).
[28]	No	mpMRI, ERSPC-RC, PBCG-RC, FPC-RC	FPC-RC was superior to mpMRI in diagnosing PCa and csPCa. Compared with PBCG-RC and ERSPC-RC, mpMRI has higher accuracy in predicting PCa, but similar performance in predicting csPCa.
[29]	Yes	mpMRI-RCs	When predicting csPCa, RC-R has a higher AUC than RC-A.
[30]	No	mpMRI, mpMRI-DW-DCE-MRSI	When diagnosing PCa, mpMRI-DW-DCE-MRSI has higher sensitivity and specificity than MRI.
[31]	No	MRI/ultrasound fusion biopsy, SB	When detecting PCa, combined biopsy provides a higher DR than SB and fusion-guided biopsy alone. When detecting csPCa, fusion-guided biopsy alone provides a higher detection rate than SB alone.
[32]	Yes	MRI- TBx, SB	When detecting PCa and csPCa, MRI-TBx provides a higher DR than SB alone.
[33]	No	${}^{68}Ga$-PSMA TPBx, mpMRI-TPBx, eSPBx	When detecting csPCa, the undetected rates of ${}^{68}Ga$-PSMA-TPBx, mpMRI-TPBx, and eSPBx were 4.5% and 18.1% and 31.8%. The accuracy rates of mpMRI-TPBx and ${}^{68}Ga$-PSMA-TPBx in the diagnosis of csPCa were 73.7% and 77.5%, respectively.

mpMRI: multiparametric magnetic resonance imaging; PCa: prostate cancer; PSA: prostate-specific antigen; csPCa: clinically significant prostate cancer; DR: detection rate; MRI-TBx: mpMRI- targeted biopsy; SB: systematic biopsy; TRUS-Bx: transrectal ultrasonography-guided biopsy; PSMA: prostate-specific membrane antigen; NPV: negative predictive value; MRI-GB: MRI-guided biopsy; TRUS-GB: transrectal ultrasound-guided systematic biopsy; micro-US: micro-ultrasonography; ERSPC-RC: European Randomized Study of Screening for Prostate Cancer Risk calculators; PBCG-RC: Prostate Biopsy Collaborative Group Risk Calculator; FPC-RC: Foggia Prostate Cancer Risk Calculator; AUC: The area under the ROC curve; mpMRI-RC: mpMRI-Risk calculator; CI: confidence interval; TPBx: targeted biopsy; mpMRI-DW-DCE-MRSI: mpMRI combination of diffusion-weighted, Dynamic contrast-enhanced and MR spectroscopic imaging; eSPBx: extended systematic prostate biopsy.

**Table 2:** Summary of feature selection methods used for radiomics analysis.

	MRI Sequence	Feature Selection	Conclusion
[60]	T2WI, DWI	Select-K-best method, LASSO	The performance of detecting clinically significant prostate cancer (csPCa) can be enhanced by using a combination of bpMRI features and the nomograph of PI-RADS.
[61]	T2WI, ADC, DWI	RF, LASSO	MRI-derived delta radiomics show performance comparable to the expert readers of prostate MRI.
[62]	T2WI, ADC, DWI, DCE-MRI	LASSO, PCA, RF	Complementary information from in-situ RS and mpMRI radiomics features allowed to accurately stratify the ISUP GG >1 ISUP GG≤1 as well as discriminate ISUP GG≥1 ISUP GG <1 sites using SVM classifiers.
[63]	T2WI, ADC, DWI	RF	In the diagnosis of PCa and recognition of P504s/P64 status, the RF algorithm model exhibits superior performance.
[64]	T2WI, DWI, DCE-MRI	LASSO	When evaluating PI-RADS 4/5 scores, the R-logistic model outperforms clinical indicators in distinguishing between benign and malignant PCa.
[65]	T2WI, ADC, DWI	RF, LASSO	ML and radiomics approach based on public datasets successfully identify clinically significant PCa.
[66]	T2WI	RF	ML classifier based on T2WI radiomic features demonstrated good performance predicting csPCa in PI-RADS 3 lesions.
[67]	ADC	PCC, PCA	Automated segmentation-based radiomics models can achieve similar effects to manual segmentation, preoperative biopsy, and PI-RADS assessment in differentiating PCa.
[68]	T2WI, ADC, DCE-MRI	LASSO, RF	ML-based bp-MRI radiomics model analysis demonstrated superior diagnostic performance to traditional PI-RADS v2.1 score in predicting PCa histological grade.
[69]	T2WI	LASSO	T2WI-based radiomic features show the potential to predict side-specific probabilities of pathological ECE status and may facilitate individualized preoperative prediction of PCa patients.
[70]	T2WI, ADC, DKI	Cross-validated	MRI radiomics features are promising markers of PCa aggressiveness on the histopathological and genomics levels.
[71]	T2WI, ADC, DKI	mRMR, LASSO	MpMRI-based radiomics features have the potential to discriminate between csPCa and ciPCa noninvasively.
[72]	DCE-MRI	k-best and LASSO	Combined analysis of the first and most vital phases of raw DCE-MRI images combining radiomics can predict PCa invasiveness.
[54]	T2WI, ADC	RF classifier model	Radiomics features can be used as non-invasive biomarkers to predict the Gleason score.

bp-MRI: biparametric MRI; csPCa: clinically significant PCa; AS: active surveillance; RS: Raman spectroscopy; SVM: support vector machines; ML: machine learning; ADC: Apparent diffusion coefficient; T2WI: T2-weighted imaging; DWI: Diffusion-weighted imaging; RF: Random forest; LASSO: least absolute shrinkage and selection operator; PCA: principal component analysis; PCC: Pearson correlation coefficient; mRMR: Max-Relevance and Min-Redundant; ciPCa: clinically insignificant PCa.

**Table 3:** Application of modeling techniques in radiomics research.

	Model	Purpose	Result
[74]	Multivariable logistic regression model	Quantitative imaging features from mpMRI were used to predict PCa	The success rate of radiomic features in PSAD and PI-RADS was 35.0% and 34.4%, respectively.
[75]	A combined radiomics model (Rad-score) and PI-RADS	To study whether the radiomics model helps improve the performance of PI-RADS v2.1 in PCa	Rad-score provides higher AUC than PI-RADS (AUC = 0.861 vs. 0.845).
[61]	Logistic regression and RF	Compare the performance of the PRECISE scoring system with several MRI-derived delta-radiomics models	RF had the most heightened sensitivity (92.6%) and NPV (92.6%) for predicting disease progression.
[76]	KNN, logistic regression, LDA, GLM, SVM, RF	To study the added value of MRI-derived radiomics features in order to improve the baseline prediction of PCa progression in AS patients	AUC value increased from 0.61 (95% CI 0.481–0.743) to 0.75 (95% CI 0.64–0.86).
[77]	New-Clinical, ComBat Combined model	Develop and internally validate a new risk prediction model for LNI in PCa	Using this model can avoid up to 80% of eLND, but it is possible to miss only 1.1% of patients with LNI.
[78]	SVM	To determine which PCa patients can safely avoid epLND by predicting LNI through a radiomics-based ML method.	The proposed IRM achieved a superior AUC of 0.915 (95% CI: 0.846–0.984) in the test set.
[79]	SVM	To evaluate the performance of combined PET and mpMRI radiomics in grouping prediction of psGSs	The combined PET + ADC radiomics provided the best overall performance (mean ± stdv 82 ± 5%).
[80]	LASSO, SVM	Develop a radiomics model that can predict csPCa	The highest performance related to DWI b2000 model with an AUC = 0.84 (95% CI, 0.63–0.90), specificity = 75%, sensitivity = 90%, and informed degree = 0.65.
[60]	Logistic regression	Develop and validate a radiologic nomogram based on multimodal MRI to predict csPCa	In the training set, test set, verification set 1, and validation set 2, the AUC of the nomograph is 0.967, 0.964, 0.945, and 0.942, respectively.

AUC: area under the receiver operator characteristic curve; PN: Parenclitic network; LASSO: Least absolute shrinkage and selection operator; RF: Random forest; PPV: positive predictive value; NPV: negative predictive value; KNN: K-nearest neighbor; LDA: Linear discriminant analysis; GLM: General linear model; SVM: Support vector machine; ML: machine learning; ROC: receiver operating characteristic curve; AS: active surveillance; CI: confidence interval; eLND: extended lymph node dissection; LNI: lymph node involvement; epLND: extended pelvic lymph node dissection; IRM: integrative radiomics model; GS: Gleason score; ECE: extracapsular extension; psGSs: postoperative Gleason scores; csPCa: clinically significant prostate cancer.

**Table 4:** Summary of radiomics stability studies.

	Stability	Purpose	Conclusion
[94]	MS and FS	To explore the potential of radiomic features in identifying GS < 7, =7 and >7	The 2D model performed better than the 3D model.
[95]	FS	To evaluate the effect of different image normalization methods on the robustness of MRI features in a multicenter.	The percentage of stable features varies from 3.4% to 8% depending on the normalization method.
[98]	MS	To assess the potential of clinic-based models, radiomics based on multiparameter ultrasound, and combined models to predict PCa.	The combined model achieved better predictive performance than the radiomics and the clinical model.
[99]	FS	To explore the stability of the radiomics features extracted from T2 weighted MR Linac images for the five common influencing factors	25 of 1409 radiomics features remained robust.
[97]	FS	The robustness of MRI radiomics features with various MRI scan protocols and scanners was investigated using an MRI radiomics model.	For reproducibility measures, average T1- and T2-weighted image ICCs were higher for phantoms than healthy volunteers.
[86]	FS	Selected Robust radiomics features with minimal variation with test-retest experiments.	Test-retest results are not generalizable.
[100]	MS	To systematically review the quality of multicenter studies on MRI radiomics for diagnosing clinically significant PCa.	CLAIM scores ranged from 71.1% to 80.6% and RQS ranged from 44.4% to 58.3%.
[101]	FS	Explores the variability of radiomics features extracted from images acquired with a 0.35 T scanner with an integrated MRI-Linac.	130 out of 1085 radiomics features showed high robustness in phantom and patient data.

MS: model stability; FS: feature stability; CI: confidence interval; GS: Gleason score; ICC: intraclass correlation coefficient; CLMAIM: checklist for artificial intelligence in medical imaging; RQS: radiomics quality score; SWE: shear-wave elastography.

**Table 5:** Summary of recent radiogenomics studies.

	Image	Gene	Purpose	Conclusion
[108]	MRI	PTEN and ERG	To determine the relationship between biomarkers PTEN and ERG with visible and invisible PCa lesions in MRI	MRI-invisible lesions had less PTEN loss and ERG-positive expression than MRI-visible lesions.
[109]	MRI	BRCA 2	To explore the potential role of prophylactic PCa resection in the primary prevention of PCa mortality.	BRCA2 carriers have a higher incidence of PCa and progress faster.
[110]	mpMRI	BRCA	To help better understand mpMRI features of BRCA- related PCa	BRCA-related PCa may have a suggestive invasive appearance on MRI before treatment.
[111]	HP${}^{13}C$-MRI	Epithelium mRNA	The role of HP${}^{13}C$-MRI in low, medium, and high-risk PCa was investigated to evaluate the differences in metabolic phenotypes of tumors.	HP${}^{13}C$-MRI can show csPCa according to the potential metabolic difference between epithelial and stromal tumor zones.
[112]	mpMRI	RNAs (snoRNA)	To understand the biological basis of PCa visibility on mpMRI.	mpMRI visibility-related nimbosus markers are associated with PCa visibility and invasiveness on mpMRI.
[113]	mpMRI	hypoxia gene signature	To determine the relationship between Ragnum-signature and hypoxia and heterogeneity within dominant (index) lesions of PCa.	The signature of the index lesion reflects hypoxia and predicts the prognosis of PCa.
[114]	mpMRI	mRNA	To more accurately and reliably identify PCa, the feasibility of combining the SelectMDx with mpMRI results was evaluated.	The combination of SelectMDx, previous biopsy history, and PI-RADS into the new scoring system resulted in significant PCa DR.
[115]	MRI	BRCA 2	To study the pathogenesis of t-SNPC or the clinical process of related genetic information.	T-SNPC is an invasive phenotype. Early detection and pathological diagnosis of gene mutations (BRCA 1/2, etc.) may improve the survival rate.
[116]	MRI	genetic profiles, RNA sequencing	To determine changes in gene expression between BPH and PCa	Genes upregulated in BPH only and downregulated in BPH/PCa, and vice versa.
[117]	MRI	RNA	To study the relationship between hyperlipidemia and PCa.	The expression characteristics of PCa genes related to hyperlipidemia identified that PCa samples had no common genetic changes - TMPRSS2-ERG fusion and PTEN deletion/mutation.
[118]	MRI	microRNA	Comprehensive integration of tissue and circulating microRNA profiles, MRI biomarkers, and clinical data for early detection of PCa.	PCa was associated with the underexpression of the miRNA profiled and normalized ADC. The overexpression of miRNAs in plasma was associated with csPCa.
[119]	bpMRI	hypoxia-related genes	Exploring the relationship between texture feature phenotype and targeted sequence RNA expression of hypoxia related genes	Changes in hypoxia related genes may lead to a decrease in the survival rate of PCa patients.
[120]	MRI	miRNA	Unraveling potential molecular interactions of PCa from T2c to T3b stages	Association between genes and miRNAs that play key roles in PCa diagnosis.
[121]	MRI	SNP(DNA)	Evaluate the effectiveness of genetic analysis in guiding PCa screening in community settings.	This study using SNP targeting PCa screening in the UK community is feasible, with an overall absorption rate of 26%.

PTEN: phosphatase and tensin homolog; ERG: ETS-related gene; BRCA: breast cancer gene; HP 13C-MRI: Hyperpolarised magnetic resonance imaging; LDH: lactate dehydrogenase; MCT: monocarboxylate transport; snoRNA: small nucleolar RNAs; DR: detection rate; GGG: Gleason grade group; t-SNPC: treatment-emergent small cell/neuroendocrine prostate cancer; BPH: benign prostatic hyperplasia; SNP: single nucleotide polymorphism.

**Table 6:** Summary of recent multi-omics studies.

	Applied Omics	Purpose	Conclusion
[141]	mRNA, miRNA, DNA methylation, CNVs, lncRNA	They have developed a novel, robust classification for understanding PCa relapse.	The DL-based model was proven robust by external validation.
[139]	Transcriptomics, metabolomics	Identify prognostic multi-omics signatures from the covered networks.	Signature 1 is significantly prognostic in the high-Gleason risk group, and signature 2 is significantly prognostic in the low-Gleason group.
[142]	mRNA, microRNA, long noncoding RNA, DNA methylation, and somatic mutation	Accurately identified specific molecular signatures and judged potential clinical outcomes from a multi-omics perspective	Identified three clusters independently of ten multi-omics integrative clustering algorithms.
[143]	Untargeted RNA sequencing, proteomics, and metabolomics	Test the feasibility of applying a multi-omics approach on an in vivo panel of paired HN and CRPC tumor models.	Metabolomics identifies increased N-acetyl aspartate (NAA) and N-Acetyl aspartyl glutamate (NAAG) in all three models of CRPC.
[144]	somatic mutations, somatic copy number alterations (SCNAs), DNA methylation, and mRNA expression	Provide a comprehensive evaluation of GPCRs expression in primary PCa	GPCRs exhibit low expression levels and mutation frequencies, which should contribute to the focus on GPCRs in oncology.
[145]	mRNA, miRNA, methylation, CNA, and SNV	Perform a multi-omics analysis to identify immune genes associated with PCa	The data point toward a role for LILRB molecules and especially LILRB1 and suggest that these receptors could play a role in the resistance of PCa to antitumor immune response.
[140]	DNA methylation	Building genetic models to predict methylation and perform association analysis with PCa risk.	759 CpG sites were identified whose predicted DNA methylation levels correlated after Bonferroni correction.
[146]	m6A methylation	To know gene expression, DNA methylation status, and CNVs for each putative m6A regulator	In 27 genes, 18 showed significant differential expression between normal and PCa samples.
[147]	mRNA, miRNA, lncRNA, DNA methylation, gene mutation	Identify and judge potential clinical outcomes based on multi-omics data	When the number of clusters is 3, the scores of the two methods are closer.

CRPC: Castration-resistant prostate cancer; GPCRs: G protein-coupled receptors; m6A: RNA N6-methyladenosine; CNVs: copy number variations; BCR: biochemical recurrence; BER: balanced error rate.

**Table 7:** Public available datasets for prostate cancer.

Data Description	Website Index
Provides information between MRI and US images for a given PCa patient.	[150,151]
250 were benign, and 250 were malignant (50 in grade 3, 100 in grade 4, and 100 in grade 5)	[152]
The multi-parametric data have been acquired from two commercial scanners (1.5 T and 3 T), including T2W, DCE, DWI, and MRSI	[44]
7756 cases of prostate MRI were included, of which 3050 cases were labeled manually	[153]
169 MRI images and 3 TRUS images	[154,155,156]
228 patients and 172 patients with T2w PCa MRI collected from TCIA	[157]
The data set of 525 patients was divided into training (n = 368, 70%), validation (n = 79, 15%), and testing (n = 78, 15%) queues	[158]
Including 299 certified PCa and 60 extracted radiomic features	[158]
Three-dimensional MRI scan of the PCa region containing 50 patients	[154]
106 clinical prostate MRI data, of which 100 MRI data were available	[154]
Examination, diagnosis, and treatment data of PCa patients. Including PSA and various biochemical data	[159]
Segmentation and labeling of the central glands and peripheral regions of the prostate	[160]
Microscopic scan of a prostate biopsy sample with imperfect labels and large images	[161]

MRI: Magnetic resonance imaging; US: ultrasound; PCa: prostate cancer; T2W: T2-weighted; DCE: dynamic contrast-enhanced; DWI: diffusion-weighted imaging; MRSI: magnetic resonance spectroscopic imaging; TCIA: the cancer imaging archive; csPCa: clinically significant prostate cancer; PSA: prostate-specific antigen.

## References

[1] Wang L., Lu B., He M., Wang Y., Wang Z., Du L. (2022). Prostate cancer incidence and mortality: Global status and temporal trends in 89 countries from 2000 to 2019. Front. Public Health.

[2] Sung H., Ferlay J., Siegel R.L., Laversanne M., Soerjomataram I., Jemal A., Bray F. (2021). Global cancer statistics 2020: GLOBOCAN estimates of incidence and mortality worldwide for 36 cancers in 185 countries. CA Cancer J. Clin..

[3] Kendrick J., Francis R., Hassan G.M., Rowshanfarzad P., Jeraj R., Kasisi C., Rusanov B., Ebert M. (2021). Radiomics for Identification and Prediction in Metastatic Prostate Cancer: A Review of Studies. Front. Oncol..

[4] Naji L., Randhawa H., Sohani Z., Dennis B., Lautenbach D., Kavanagh O., Bawor M., Banfield L., Profetto J. (2018). Digital rectal examination for prostate cancer screening in primary care: A systematic review and meta-analysis. Ann. Fam. Med..

[5] Kim J.H., Hong S.K. (2021). Clinical utility of current biomarkers for prostate cancer detection. Investig. Clin. Urol..

[6] Swanson G.P., Trevathan S., Hammonds K.A., Speights V., Hermans M.R. (2021). Gleason score evolution and the effect on prostate cancer outcomes. Am. J. Clin. Pathol..

[7] Montironi R., Cheng L., Cimadamore A., Mazzucchelli R., Scarpelli M., Santoni M., Massari F., Lopez-Beltran A. (2021). Narrative review of prostate cancer grading systems: Will the Gleason scores be replaced by the Grade Groups?. Transl. Androl. Urol..

[8] Ghafoor S., Burger I.A., Vargas A.H. (2019). Multimodality imaging of prostate cancer. J. Nucl. Med..

[9] Caglic I., Barrett T. (2019). Optimising prostate mpMRI: Prepare for success. Clin. Radiol..

[10] Scapicchio C., Gabelloni M., Barucci A., Cioni D., Saba L., Neri E. (2021). A deep look into radiomics. La Radiol. Med..

[11] Parekh V.S., Jacobs M.A. (2019). Deep learning and radiomics in precision medicine. Expert Rev. Precis. Med. Drug Dev..

[12] Gelezhe P.B., Blokhin I.A., Semenov S.S., Caruso D., Damiano C. (2021). Magnetic resonance imaging radiomics in prostate cancer radiology: What is currently known?. Digit. Diagn..

[13] Campello V.M., Martin-Isla C., Izquierdo C., Guala A., Palomares J.F.R., Vilades D., Descalzo M.L., Karakas M., Cavus E., Raisi-Estabragh Z. (2022). Minimising multi-centre radiomics variability through image normalisation: A pilot study. Sci. Rep..

[14] Eklund M., Jäderling F., Discacciati A., Bergman M., Annerstedt M., Aly M., Glaessgen A., Carlsson S., Grönberg H., Nordström T. (2021). MRI-targeted or standard biopsy in prostate cancer screening. N. Engl. J. Med..

[15] Kasivisvanathan V., Rannikko A.S., Borghi M., Panebianco V., Mynderse L.A., Vaarala M.H., Briganti A., Budäus L., Hellawell G., Hindley R.G. (2018). MRI-Targeted or Standard Biopsy for Prostate-Cancer Diagnosis. N. Engl. J. Med..

[16] Barentsz J.O., Richenberg J., Clements R., Choyke P., Verma S., Villeirs G., Rouviere O., Logager V., Fütterer J.J. (2012). ESUR prostate MR guidelines 2012. Eur. Radiol..

[17] Weinreb J.C., Barentsz J.O., Choyke P.L., Cornud F., Haider M.A., Macura K.J., Margolis D., Schnall M.D., Shtern F., Tempany C.M. (2016). PI-RADS prostate imaging–reporting and data system: 2015, version 2. Eur. Urol..

[18] Barrett T., Rajesh A., Rosenkrantz A.B., Choyke P.L., Turkbey B. (2019). PI-RADS version 2.1: One small step for prostate MRI. Clin. Radiol..

[19] Bardis M.D., Houshyar R., Chang P.D., Ushinsky A., Glavis-Bloom J., Chahine C., Bui T.L., Rupasinghe M., Filippi C.G., Chow D.S. (2020). Applications of artificial intelligence to prostate multiparametric MRI (mpMRI): Current and emerging trends. Cancers.

[20] Chiacchio G., Castellani D., Nedbal C., De Stefano V., Brocca C., Tramanzoli P., Galosi A.B., Donalisio da Silva R., Teoh J.Y.C., Tiong H.Y. (2023). Radiomics vs radiologist in prostate cancer. Results from a systematic review. World J. Urol..

[21] Grey A.D., Scott R., Shah B., Acher P., Liyanage S., Pavlou M., Omar R., Chinegwundoh F., Patki P., Shah T.T. (2022). Multiparametric ultrasound versus multiparametric MRI to diagnose prostate cancer (CADMUS): A prospective, multicentre, paired-cohort, confirmatory study. Lancet Oncol..

[22] Klotz L., Chin J., Black P.C., Finelli A., Anidjar M., Bladou F., Mercado A., Levental M., Ghai S., Chang S.D. (2021). Comparison of multiparametric magnetic resonance imaging–targeted biopsy with systematic transrectal ultrasonography biopsy for biopsy-naive men at risk for prostate cancer: A phase 3 randomized clinical trial. JAMA Oncol..

[23] Bang S., Yu J., Chung J.H., Song W., Kang M., Sung H.H., Jeon H.G., Jeong B.C., Seo S.I., Lee H.M. (2021). Usefulness of MRI targeted prostate biopsy for detecting clinically significant prostate cancer in men with low prostate-specific antigen levels. Sci. Rep..

[24] Bass E.J., Pantovic A., Connor M.J., Loeb S., Rastinehad A.R., Winkler M., Gabe R., Ahmed H.U. (2022). Diagnostic accuracy of magnetic resonance imaging targeted biopsy techniques compared to transrectal ultrasound guided biopsy of the prostate: A systematic review and meta-analysis. Prostate Cancer Prostatic Dis..

[25] Del Monte M., Cipollari S., Del Giudice F., Pecoraro M., Bicchetti M., Messina E., Dehghanpour A., Ciardi A., Sciarra A., Catalano C. (2022). MRI-directed biopsy for primary detection of prostate cancer in a population of 223 men: MRI In-Bore vs MRI-transrectal ultrasound fusion-targeted techniques. Br. J. Radiol..

[26] Emmett L., Buteau J., Papa N., Moon D., Thompson J., Roberts M.J., Rasiah K., Pattison D.A., Yaxley J., Thomas P. (2021). The additive diagnostic value of prostate-specific membrane antigen positron emission tomography computed tomography to multiparametric magnetic resonance imaging triage in the diagnosis of prostate cancer (PRIMARY): A prospective multicentre study. Eur. Urol..

[27] Hofbauer S.L., Luger F., Harland N., Plage H., Reimann M., Hollenbach M., Gusenleitner A., Stenzl A., Schlomm T., Wiemer L. (2022). A non-inferiority comparative analysis of micro-ultrasonography and MRI-targeted biopsy in men at risk of prostate cancer. BJU Int..

[28] Falagario U.G., Silecchia G., Bruno S.M., Di Nauta M., Auciello M., Sanguedolce F., Milillo P., Macarini L., Selvaggio O., Carrieri G. (2021). Does multiparametric magnetic resonance of prostate outperform risk calculators in predicting prostate cancer in biopsy naïve patients?. Front. Oncol..

[29] Pallauf M., Steinkohl F., Zimmermann G., Horetzky M., Rajwa P., Pradere B., Lindner A.K., Pichler R., Kunit T., Shariat S.F. (2022). External validation of two mpMRI-risk calculators predicting risk of prostate cancer before biopsy. World J. Urol..

[30] Ahmed I.H.A.E., Mohamed Ali Hassan H.G.E., Abo ElMaaty M.E.G., ElDaisty El Metwally S.E.M. (2022). Role of MRI in diagnosis of prostate cancer and correlation of results with transrectal ultrasound guided biopsy “TRUS”. Egypt. J. Radiol. Nucl. Med..

[31] Dorfinger J., Ponholzer A., Stolzlechner M., Lenart S., Baltzer P., Toepker M. (2022). MRI/ultrasound fusion biopsy of the prostate compared to systematic prostate biopsy—Effectiveness and accuracy of a combined approach in daily clinical practice. Eur. J. Radiol..

[32] Lenfant L., Renard-Penna R., de Rycke Y., Rouprêt M., Beaugerie A., Comperat E., Chartier-Kastler E., Mozer P.C. (2022). Dynamic evaluation of MRI-targeted, systematic and combined biopsy for prostate cancer diagnosis through 10 years of practice in a single institution. World J. Urol..

[33] Pepe P., Pepe L., Cosentino S., Ippolito M., Pennisi M., Fraggetta F. (2022). Detection Rate of 68Ga-PSMA PET/CT vs. mpMRI Targeted Biopsy for Clinically Significant Prostate Cancer. Anticancer Res..

[34] O’Connor L., Wang A., Walker S.M., Yerram N., Pinto P.A., Turkbey B. (2020). Use of multiparametric magnetic resonance imaging (mpMRI) in localized prostate cancer. Expert Rev. Med. Devices.

[35] Cuocolo R., Cipullo M.B., Stanzione A., Ugga L., Romeo V., Radice L., Brunetti A., Imbriaco M. (2019). Machine learning applications in prostate cancer magnetic resonance imaging. Eur. Radiol. Exp..

[36] Li H., Lee C.H., Chia D., Lin Z., Huang W., Tan C.H. (2022). Machine Learning in Prostate MRI for Prostate Cancer: Current Status and Future Opportunities. Diagnostics.

[37] Huang X., Zhang B., Zhang X., Tang M., Miao Q., Li T., Jia G. (2021). Application of U-Net based multiparameter magnetic resonance image fusion in the diagnosis of prostate cancer. IEEE Access.

[38] Soerensen S.J.C., Fan R.E., Seetharaman A., Chen L., Shao W., Bhattacharya I., Kim Y.h., Sood R., Borre M., Chung B.I. (2021). Deep learning improves speed and accuracy of prostate gland segmentations on magnetic resonance imaging for targeted biopsy. J. Urol..

[39] Panebianco V., Barchetti G., Simone G., Del Monte M., Ciardi A., Grompone M.D., Campa R., Indino E.L., Barchetti F., Sciarra A. (2018). Negative multiparametric magnetic resonance imaging for prostate cancer: What’s next?. Eur. Urol..

[40] Ferro M., de Cobelli O., Musi G., del Giudice F., Carrieri G., Busetto G.M., Falagario U.G., Sciarra A., Maggi M., Crocetto F. (2022). Radiomics in prostate cancer: An up-to-date review. Ther. Adv. Urol..

[41] Tombal B., Rezazadeh A., Therasse P., Van Cangh P.J., Vande Berg B., Lecouvet F.E. (2005). Magnetic resonance imaging of the axial skeleton enables objective measurement of tumor response on prostate cancer bone metastases. Prostate.

[42] Quantitative Imaging Biomarkers Alliance. https://www.rsna.org/research/quantitative-imaging-biomarkers-alliance.

[43] Image Biomarker Standardisation Initiative. https://arxiv.org/abs/1612.07003.

[44] European Imaging Biomarkers Alliance. https://www.myesr.org/research/european-imaging-biomarkers-alliance-eiball.

[45] Onofrey J.A., Casetti-Dinescu D.I., Lauritzen A.D., Sarkar S., Venkataraman R., Fan R.E., Sonn G.A., Sprenkle P.C., Staib L.H., Papademetris X. (2019). Generalizable multi-site training and testing of deep neural networks using image normalization. Proceedings of the 2019 IEEE 16th International Symposium on Biomedical Imaging (ISBI 2019).

[46] Anand L., Mewada S., Shamsi W., Ritonga M., Aflisia N., KumarSarangi P., NdoleArthur M. (2023). Diagnosis of Prostate Cancer Using GLCM Enabled KNN Technique by Analyzing MRI Images. BioMed Res. Int..

[47] Baeßler B., Weiss K., Dos Santos D.P. (2019). Robustness and reproducibility of radiomics in magnetic resonance imaging: A phantom study. Investig. Radiol..

[48] Gillies R.J., Kinahan P.E., Hricak H. (2016). Radiomics: Images Are More than Pictures, They Are Data. Radiology.

[49] Gunashekar D.D., Bielak L., Hägele L., Oerther B., Benndorf M., Grosu A.L., Brox T., Zamboglou C., Bock M. (2022). Explainable AI for CNN-based prostate tumor segmentation in multi-parametric MRI correlated to whole mount histopathology. Radiat. Oncol..

[50] Wang Z., Wu R., Xu Y., Liu Y., Chai R., Ma H. (2023). A two-stage CNN method for MRI image segmentation of prostate with lesion. Biomed. Signal Process. Control.

[51] Jimenez-Pastor A., Lopez-Gonzalez R., Fos-Guarinos B., Garcia-Castro F., Wittenberg M., Torregrosa-Andrés A., Marti-Bonmati L., Garcia-Fontes M., Duarte P., Gambini J.P. (2023). Automated prostate multi-regional segmentation in magnetic resonance using fully convolutional neural networks. Eur. Radiol..

[52] Song E., Long J., Ma G., Liu H., Hung C.C., Jin R., Wang P., Wang W. (2023). Prostate lesion segmentation based on a 3D end-to-end convolution neural network with deep multi-scale attention. Magn. Reson. Imaging.

[53] Salvi M., De Santi B., Pop B., Bosco M., Giannini V., Regge D., Molinari F., Meiburger K.M. (2022). Integration of Deep Learning and Active Shape Models for More Accurate Prostate Segmentation in 3D MR Images. J. Imaging.

[54] Chaddad A., Niazi T., Probst S., Bladou F., Anidjar M., Bahoric B. (2018). Predicting Gleason Score of Prostate Cancer Patients Using Radiomic Analysis. Front. Oncol..

[55] Chaddad A., Kucharczyk M.J., Niazi T. (2018). Multimodal radiomic features for the predicting gleason score of prostate cancer. Cancers.

[56] Chaddad A., Kucharczyk M.J., Desrosiers C., Okuwobi I.P., Katib Y., Zhang M., Rathore S., Sargos P., Niazi T. (2020). Deep radiomic analysis to predict gleason score in prostate cancer. IEEE Access.

[57] Tharmaseelan H., Rotkopf L.T., Ayx I., Hertel A., Nörenberg D., Schoenberg S.O., Froelich M.F. (2022). Evaluation of radiomics feature stability in abdominal monoenergetic photon counting CT reconstructions. Sci. Rep..

[58] Zebari R., Abdulazeez A., Zeebaree D., Zebari D., Saeed J. (2020). A comprehensive review of dimensionality reduction techniques for feature selection and feature extraction. J. Appl. Sci. Technol. Trends.

[59] Blum A.L., Langley P. (1997). Selection of relevant features and examples in machine learning. Artif. Intell..

[60] Jing G., Xing P., Li Z., Ma X., Lu H., Shao C., Lu Y., Lu J., Shen F. (2022). Prediction of clinically significant prostate cancer with a multimodal MRI-based radiomics nomogram. Front. Oncol..

[61] Sushentsev N., Rundo L., Blyuss O., Nazarenko T., Suvorov A., Gnanapragasam V.J., Sala E., Barrett T. (2022). Comparative performance of MRI-derived PRECISE scores and delta-radiomics models for the prediction of prostate cancer progression in patients on active surveillance. Eur. Radiol..

[62] Grajales D., Picot F., Shams R., Dallaire F., Sheehy G., Alley S., Barkati M., Delouya G., Carrier J.F., Birlea M. (2022). Image-guided Raman spectroscopy navigation system to improve transperineal prostate cancer detection. Part 2: In-vivo tumor-targeting using a classification model combining spectral and MRI-radiomics features. J. Biomed. Opt..

[63] Liu Y.F., Shu X., Qiao X.F., Ai G.Y., Liu L., Liao J., Qian S., He X.J. (2022). Radiomics-based machine learning models for predicting P504s/P63 immunohistochemical expression: A noninvasive diagnostic tool for prostate cancer. Front. Oncol..

[64] Ma L., Zhou Q., Yin H., Ang X., Li Y., Xie G., Li G. (2022). Texture analysis based on PI-RADS 4/5-scored magnetic resonance images combined with machine learning to distinguish benign lesions from prostate cancer. Transl. Cancer Res..

[65] Donisi L., Cesarelli G., Castaldo A., De Lucia D.R., Nessuno F., Spadarella G., Ricciardi C. (2021). A Combined Radiomics and Machine Learning Approach to Distinguish Clinically Significant Prostate Lesions on a Publicly Available MRI Dataset. J. Imaging.

[66] Hectors S.J., Chen C., Chen J., Wang J., Gordon S., Yu M., Al Hussein Al Awamlh B., Sabuncu M.R., Margolis D.J., Hu J.C. (2021). Magnetic Resonance Imaging Radiomics-Based Machine Learning Prediction of Clinically Significant Prostate Cancer in Equivocal PI-RADS 3 Lesions. J. Magn. Reson. Imaging.

[67] Han C., Ma S., Liu X., Liu Y., Li C., Zhang Y., Zhang X., Wang X. (2021). Radiomics Models Based on Apparent Diffusion Coefficient Maps for the Prediction of High-Grade Prostate Cancer at Radical Prostatectomy: Comparison With Preoperative Biopsy. J. Magn. Reson. Imaging.

[68] Zhang L., Zhe X., Tang M., Zhang J., Ren J., Zhang X., Li L. (2021). Predicting the Grade of Prostate Cancer Based on a Biparametric MRI Radiomics Signature. Contrast Media Mol. Imaging.

[69] Ma S., Xie H., Wang H., Yang J., Han C., Wang X., Zhang X. (2020). Preoperative prediction of extracapsular extension: Radiomics signature based on magnetic resonance imaging to stage prostate cancer. Mol. Imaging Biol..

[70] Hectors S.J., Cherny M., Yadav K.K., Beksaç A.T., Thulasidass H., Lewis S., Davicioni E., Wang P., Tewari A.K., Taouli B. (2019). Radiomics Features Measured with Multiparametric Magnetic Resonance Imaging Predict Prostate Cancer Aggressiveness. J. Urol..

[71] (2019). Multi-parametric MRI-based radiomics signature for discriminating between clinically significant and insignificant prostate cancer: Cross-validation of a machine learning method. Eur. J. Radiol..

[72] Liu B., Cheng J., Guo D.J., He X.J., Luo Y.D., Zeng Y., Li C.M. (2019). Prediction of prostate cancer aggressiveness with a combination of radiomics and machine learning-based analysis of dynamic contrast-enhanced MRI. Clin. Radiol..

[73] Spohn S.K., Bettermann A.S., Bamberg F., Benndorf M., Mix M., Nicolay N.H., Fechter T., Hölscher T., Grosu R., Chiti A. (2021). Radiomics in prostate cancer imaging for a personalized treatment approach-current aspects of methodology and a systematic review on validated studies. Theranostics.

[74] Ogbonnaya C.N., Zhang X., Alsaedi B.S., Pratt N., Zhang Y., Johnston L., Nabi G. (2021). Prediction of Clinically Significant Cancer Using Radiomics Features of Pre-Biopsy of Multiparametric MRI in Men Suspected of Prostate Cancer. Cancers.

[75] Li M., Yang L., Yue Y., Xu J., Huang C., Song B. (2021). Use of radiomics to improve diagnostic performance of PI-RADS v2. 1 in prostate cancer. Front. Oncol..

[76] Sushentsev N., Rundo L., Blyuss O., Gnanapragasam V.J., Sala E., Barrett T. (2021). MRI-derived radiomics model for baseline prediction of prostate cancer progression on active surveillance. Sci. Rep..

[77] Bourbonne V., Jaouen V., Nguyen T.A., Tissot V., Doucet L., Hatt M., Visvikis D., Pradier O., Valéri A., Fournier G. (2021). Development of a Radiomic-Based Model Predicting Lymph Node Involvement in Prostate Cancer Patients. Cancers.

[78] Zheng H., Miao Q., Liu Y., Mirak S.A., Hosseiny M., Scalzo F., Raman S.S., Sung K. (2022). Multiparametric MRI-based radiomics model to predict pelvic lymph node invasion for patients with prostate cancer. Eur. Radiol..

[79] Solari E.L., Gafita A., Schachoff S., Bogdanović B., Villagrán Asiares A., Amiel T., Hui W., Rauscher I., Visvikis D., Maurer T. (2022). The added value of PSMA PET/MR radiomics for prostate cancer staging. Eur. J. Nucl. Med. Mol. Imaging.

[80] Bevilacqua A., Mottola M., Ferroni F., Rossi A., Gavelli G., Barone D. (2021). The primacy of high B-value 3T-DWI radiomics in the prediction of clinically significant prostate cancer. Diagnostics.

[81] Kalendralis P. (2022). Artificial Intelligence Applications in Radiotherapy: The Role of the FAIR Data Principles.

[82] Cysouw M.C., Jansen B.H., van de Brug T., Oprea-Lager D.E., Pfaehler E., de Vries B.M., van Moorselaar R.J., Hoekstra O.S., Vis A.N., Boellaard R. (2021). Machine learning-based analysis of [18F] DCFPyL PET radiomics for risk stratification in primary prostate cancer. Eur. J. Nucl. Med. Mol. Imaging.

[83] Guglielmo P., Marturano F., Bettinelli A., Gregianin M., Paiusco M., Evangelista L. (2021). Additional Value of PET Radiomic Features for the Initial Staging of Prostate Cancer: A Systematic Review from the Literature. Cancers.

[84] Li L., Shiradkar R., Leo P., Algohary A., Fu P., Tirumani S.H., Mahran A., Buzzy C., Obmann V.C., Mansoori B. (2021). A novel imaging based Nomogram for predicting post-surgical biochemical recurrence and adverse pathology of prostate cancer from pre-operative bi-parametric MRI. EBioMedicine.

[85] Wang S., Lin C., Kolomaya A., Ostdiek-Wille G.P., Wong J., Cheng X., Lei Y., Liu C. (2022). Compute Tomography Radiomics Analysis on Whole Pancreas Between Healthy Individual and Pancreatic Ductal Adenocarcinoma Patients: Uncertainty Analysis and Predictive Modeling. Technol. Cancer Res. Treat..

[86] van Timmeren J.E., Leijenaar R.T., van Elmpt W., Wang J., Zhang Z., Dekker A., Lambin P. (2016). Test–retest data for radiomics feature stability analysis: Generalizable or study-specific?. Tomography.

[87] Vandewinckele L., Claessens M., Dinkla A., Brouwer C., Crijns W., Verellen D., van Elmpt W. (2020). Overview of artificial intelligence-based applications in radiotherapy: Recommendations for implementation and quality assurance. Radiother. Oncol..

[88] Castillo T J.M., Starmans M.P., Arif M., Niessen W.J., Klein S., Bangma C.H., Schoots I.G., Veenland J.F. (2021). A multi-center, multi-vendor study to evaluate the generalizability of a radiomics model for classifying prostate cancer: High grade vs. low grade. Diagnostics.

[89] Rajagopal A., Redekop E., Kemisetti A., Kulkarni R., Raman S., Sarma K., Magudia K., Arnold C.W., Larson P.E. (2023). Federated learning with research prototypes: Application to multi-center MRI-based detection of prostate cancer with diverse histopathology. Acad. Radiol..

[90] Chaddad A., Lu Q., Li J., Katib Y., Kateb R., Tanougast C., Bouridane A., Abdulkadir A. (2023). Explainable, domain-adaptive, and federated artificial intelligence in medicine. IEEE/CAA J. Autom. Sin..

[91] Scalco E., Rizzo G., Mastropietro A. (2022). The stability of oncologic MRI radiomic features and the potential role of deep learning: A review. Phys. Med. Biol..

[92] Peerlings J., Woodruff H.C., Winfield J.M., Ibrahim A., Van Beers B.E., Heerschap A., Jackson A., Wildberger J.E., Mottaghy F.M., DeSouza N.M. (2019). Stability of radiomics features in apparent diffusion coefficient maps from a multi-centre test-retest trial. Sci. Rep..

[93] Singhal N., Soni S., Bonthu S., Chattopadhyay N., Samanta P., Joshi U., Jojera A., Chharchhodawala T., Agarwal A., Desai M. (2022). A deep learning system for prostate cancer diagnosis and grading in whole slide images of core needle biopsies. Sci. Rep..

[94] Gong L., Xu M., Fang M., He B., Li H., Fang X., Dong D., Tian J. (2022). The potential of prostate gland radiomic features in identifying the Gleason score. Comput. Biol. Med..

[95] Saltybaeva N., Tanadini-Lang S., Vuong D., Burgermeister S., Mayinger M., Bink A., Andratschke N., Guckenberger M., Bogowicz M. (2022). Robustness of radiomic features in magnetic resonance imaging for patients with glioblastoma: Multi-center study. Phys. Imaging Radiat. Oncol..

[96] Gallivanone F., D’Ambrosio D., Carne I., D’Arcangelo M., Montagna P., Giroletti E., Poggi P., Vellani C., Moro L., Castiglioni I. (2022). A tri-modal tissue-equivalent anthropomorphic phantom for PET, CT and multi-parametric MRI radiomics. Phys. Med..

[97] Lee J., Steinmann A., Ding Y., Lee H., Owens C., Wang J., Yang J., Followill D., Ger R., MacKin D. (2021). Radiomics feature robustness as measured using an MRI phantom. Sci. Rep..

[98] Liang L., Zhi X., Sun Y., Li H., Wang J., Xu J., Guo J. (2021). A Nomogram based on a multiparametric ultrasound radiomics model for discrimination between malignant and benign prostate lesions. Front. Oncol..

[99] Sun M., Baiyasi A., Liu X., Shi X., Li X., Zhu J., Yin Y., Hu J., Li Z., Li B. (2022). Robustness and reproducibility of radiomics in T2 weighted images from magnetic resonance image guided linear accelerator in a phantom study. Phys. Med..

[100] Bleker J., Kwee T.C., Yakar D. (2022). Quality of Multicenter Studies Using MRI Radiomics for Diagnosing Clinically Significant Prostate Cancer: A Systematic Review. Life.

[101] Ericsson-Szecsenyi R., Zhang G., Redler G., Feygelman V., Rosenberg S., Latifi K., Ceberg C., Moros E.G. (2022). Robustness Assessment of Images From a 0.35T Scanner of an Integrated MRI-Linac: Characterization of Radiomics Features in Phantom and Patient Data. Technol. Cancer Res. Treat..

[102] Giannini V., Mazzetti S., Defeudis A., Stranieri G., Calandri M., Bollito E., Bosco M., Porpiglia F., Manfredi M., Pascale A.D. (2021). A Fully Automatic Artificial Intelligence System Able to Detect and Characterize Prostate Cancer Using Multiparametric MRI: Multicenter and Multi-Scanner Validation. Front. Oncol..

[103] Bhattacharya I., Seetharaman A., Kunder C., Shao W., Chen L.C., Soerensen S.J., Wang J.B., Teslovich N.C., Fan R.E., Ghanouni P. (2022). Selective identification and localization of indolent and aggressive prostate cancers via CorrSigNIA: An MRI-pathology correlation and deep learning framework. Med. Image Anal..

[104] Bhattacharya I., Khandwala Y.S., Vesal S., Shao W., Yang Q., Soerensen S.J., Fan R.E., Ghanouni P., Kunder C.A., Brooks J.D. (2022). A review of artificial intelligence in prostate cancer detection on imaging. Ther. Adv. Urol..

[105] Mytsyk Y., Borzhiyevs’kyy A., Kobilnyk Y., Shulyak A., Dutka I., Borzhiyevs’kyy O., Górecki A. (2022). Personalized management of prostate cancer: From molecular and imaging markers to radiogenomics. Pol. J. Radiol..

[106] Giri V.N., Knudsen K.E., Kelly W.K., Cheng H.H., Cooney K.A., Cookson M.S., Dahut W., Weissman S., Soule H.R., Petrylak D.P. (2020). Implementation of germline testing for prostate cancer: Philadelphia Prostate Cancer Consensus Conference 2019. J. Clin. Oncol..

[107] Lyu F., Li Y., Yan Z., He Q., Cheng L., Zhang P., Liu B., Liu C., Song Y., Xing Y. (2022). Identification of ISG15 and ZFP36 as novel hypoxia-and immune-related gene signatures contributing to a new perspective for the treatment of prostate cancer by bioinformatics and experimental verification. J. Transl. Med..

[108] Eineluoto J.T., Sandeman K., Pohjonen J., Sopyllo K., Nordling S., Sturenberg C., Malen A., Kilpelainen T.P., Santti H., Petas A. (2021). Associations of PTEN and ERG with magnetic resonance imaging visibility and assessment of non–organ-confined pathology and biochemical recurrence after radical prostatectomy. Eur. Urol. Focus.

[109] Tiwari R., Clark R., Fleshner N. (2022). The role of prophylactic prostatectomy as a primary prevention strategy in high-risk germline mutation carriers. Curr. Opin. Urol..

[110] Kamal O., Foster B.R., Young D.J., Hansel D.E., Coakley F.V. (2022). MRI appearance of BRCA-associated prostate cancer. Clin. Imaging.

[111] Sushentsev N., McLean M.A., Warren A.Y., Benjamin A.J., Brodie C., Frary A., Gill A.B., Jones J., Kaggie J.D., Lamb B.W. (2022). Hyperpolarised 13C-MRI identifies the emergence of a glycolytic cell population within intermediate-risk human prostate cancer. Nat. Commun..

[112] Khoo A., Liu L.Y., Sadun T.Y., Salmasi A., Pooli A., Felker E., Houlahan K.E., Ignatchenko V., Raman S.S., Sisk A.E. (2022). Prostate cancer multiparametric magnetic resonance imaging visibility is a tumor-intrinsic phenomena. J. Hematol. Oncol..

[113] Salberg U.B., Skingen V.E., Fjeldbo C.S., Hompland T., Ragnum H.B., Vlatkovic L., Hole K.H., Seierstad T., Lyng H. (2022). A prognostic hypoxia gene signature with low heterogeneity within the dominant tumour lesion in prostate cancer patients. Br. J. Cancer.

[114] Katzendorn O., von Klot C.A., Mahjoub S., Faraj Tabrizi P., Harke N.N., Tezval H., Hellms S., Hennenlotter J., Baig M.S., Stenzl A. (2022). Combination of PI-RADS score and mRNA urine test—A novel scoring system for improved detection of prostate cancer. PLoS ONE.

[115] Naiki T., Naiki-Ito A., Kawai T., Komatsu H., Nishikawa R., Gonda M., Aoki M., Sugiyama Y., Tasaki Y., Yasui T. (2022). A case of metastatic treatment-emergent small cell/neuroendocrine prostate cancer with BRCA2 mutation diagnosed by liver biopsy. IJU Case Rep..

[116] Gozal N.B., Li T., Zhang B., Nottingham C., Andriole G., Mahajan K., Kim E. (2022). PD46-06 symptomatic benign prostatic hyperplasia with immune-enriched landscapes show lower incidence of prostate cancer development. J. Urol..

[117] Han H.H., Sung J.S., Song D.W., Park C.K., Cho N.H., Choi Y.D., Ko W.J. (2022). Hyperlipidemia promotes aggressive variant prostate cancer via RNA-binding protein Quaking. Cancer Res..

[118] Panebianco V., Paci P., Pecoraro M., Conte F., Carnicelli G., Besharat Z.M., Catanzaro G., Splendiani E., Sciarra A., Farina L. (2021). Network Analysis Integrating microRNA Expression Profiling with MRI Biomarkers and Clinical Data for Prostate Cancer Early Detection: A Proof of Concept Study. Biomedicines.

[119] Ogbonnaya C.N., Alsaedi B.S., Alhussaini A.J., Hislop R., Pratt N., Nabi G. (2023). Radiogenomics Reveals Correlation between Quantitative Texture Radiomic Features of Biparametric MRI and Hypoxia-Related Gene Expression in Men with Localised Prostate Cancer. J. Clin. Med..

[120] Fischer S., Tahoun M., Klaan B., Thierfelder K.M., Weber M.A., Krause B.J., Hakenberg O., Fuellen G., Hamed M. (2019). A radiogenomic approach for decoding molecular mechanisms underlying tumor progression in prostate cancer. Cancers.

[121] Benafif S., Ni Raghallaigh H., McGrowder E., Saunders E.J., Brook M.N., Saya S., Rageevakumar R., Wakerell S., James D., Chamberlain A. (2022). The BARCODE1 Pilot: A feasibility study of using germline single nucleotide polymorphisms to target prostate cancer screening. BJU Int..

[122] Banerjee V., Wang S., Drescher M., Russell R., Siddiqui M.M. (2022). Radiogenomics influence on the future of prostate cancer risk stratification. Ther. Adv. Urol..

[123] Rachel M., Debanjan H., Sina B., Ariana F., Hannah A., Sherjeel A., Arastoo V., Phillip S., Adam R., Christos D. (2022). Radiomics and radiogenomics in pediatric neuro-oncology: A review. Neuro-Oncol. Adv..

[124] Dlamini Z., Skepu A., Kim N., Mkhabele M., Khanyile R., Molefi T., Mbatha S., Setlai B., Mulaudzi T., Mabongo M. (2022). AI and precision oncology in clinical cancer genomics: From prevention to targeted cancer therapies-an outcomes based patient care. Inform. Med. Unlocked.

[125] Sukhadia S.S., Tyagi A., Venkataraman V., Mukherjee P., Prathosh A., Divate M.D., Gevaert O., Nagaraj S.H. (2021). ImaGene: A web-based software platform for tumor radiogenomic evaluation and reporting. bioRxiv.

[126] Mardis E.R. (2013). Next-generation sequencing platforms. Annu. Rev. Anal. Chem..

[127] Li S., Tollefsbol T.O. (2021). DNA methylation methods: Global DNA methylation and methylomic analyses. Methods.

[128] Taylor B.C., Young N.L. (2021). Combinations of histone post-translational modifications. Biochem. J..

[129] Taavitsainen S., Engedal N., Cao S., Handle F., Erickson A., Prekovic S., Wetterskog D., Tolonen T., Vuorinen E., Kiviaho A. (2021). Single-cell ATAC and RNA sequencing reveal pre-existing and persistent cells associated with prostate cancer relapse. Nat. Commun..

[130] Guo W., Li L., He J., Liu Z., Han M., Li F., Xia X., Zhang X., Zhu Y., Wei Y. (2020). Single-cell transcriptomics identifies a distinct luminal progenitor cell type in distal prostate invagination tips. Nat. Genet..

[131] Yan Y., Yeon S.Y., Qian C., You S., Yang W. (2021). On the road to accurate protein biomarkers in prostate cancer diagnosis and prognosis: Current status and future advances. Int. J. Mol. Sci..

[132] Eskra J.N., Rabizadeh D., Pavlovich C.P., Catalona W.J., Luo J. (2019). Approaches to urinary detection of prostate cancer. Prostate Cancer Prostatic Dis..

[133] Chen Z., Li Z., Li H., Jiang Y. (2019). Metabolomics: A promising diagnostic and therapeutic implement for breast cancer. OncoTargets Ther..

[134] Lima A.R., Carvalho M., Aveiro S.S., Melo T., Domingues M., Macedo-Silva C., Coimbra N., Jeronimo C., Henrique R., Bastos M.d.L. (2021). Comprehensive metabolomics and lipidomics profiling of prostate cancer tissue reveals metabolic dysregulations associated with disease development. J. Proteome Res..

[135] Karczewski K.J., Snyder M.P. (2018). Integrative omics for health and disease. Nat. Rev. Genet..

[136] Das T., Andrieux G., Ahmed M., Chakraborty S. (2020). Integration of online omics-data resources for cancer research. Front. Genet..

[137] Zhang E., Zhang M., Shi C., Sun L., Shan L., Zhang H., Song Y. (2020). An overview of advances in multi-omics analysis in prostate cancer. Life Sci..

[138] Gomez-Cebrian N., Poveda J.L., Pineda-Lucena A., Puchades-Carrasco L. (2022). Metabolic phenotyping in prostate cancer using multi-omics approaches. Cancers.

[139] Xu Z., Omar M., Benedetti E., Rosenthal J., Umeton R., Krumsiek J., Pomerantz M., Imada E., Loda M., Marchionni L. (2022). Multiomics biomarkers aid prostate cancer prognostication. bioRxiv.

[140] Wu L., Yang Y., Guo X., Shu X.O., Cai Q., Shu X., Li B., Tao R., Wu C., Nikas J.B. (2020). An integrative multi-omics analysis to identify candidate DNA methylation biomarkers related to prostate cancer risk. Nat. Commun..

[141] Wei Z., Han D., Zhang C., Wang S., Liu J., Chao F., Song Z., Chen G. (2022). Deep Learning-Based Multi-Omics Integration Robustly Predicts Relapse in Prostate Cancer. Front. Oncol..

[142] Meng J., Lu X., Jin C., Zhou Y., Ge Q., Zhou J., Hao Z., Yan F., Zhang M., Liang C. (2021). Integrated multi-omics data reveals the molecular subtypes and guides the androgen receptor signalling inhibitor treatment of prostate cancer. Clin. Transl. Med..

[143] Salji M.J., Blomme A., Däbritz J.H.M., Repiscak P., Lilla S., Patel R., Sumpton D., van den Broek N.J., Daly R., Zanivan S. (2022). Multi-omics & pathway analysis identify potential roles for tumor N-acetyl aspartate accumulation in murine models of castration-resistant prostate cancer. iScience.

[144] Li S., Chen J., Chen X., Yu J., Guo Y., Li M., Pu X. (2022). Therapeutic and prognostic potential of GPCRs in prostate cancer from multi-omics landscape. Front. Pharmacol..

[145] Vittrant B., Bergeron A., Molina O.E., Leclercq M., Legare X.P., Hovington H., Picard V., Martin-Magniette M.L., Livingstone J., Boutros P.C. (2020). Immune-focused multi-omics analysis of prostate cancer: Leukocyte Ig-Like receptors are associated with disease progression. Oncoimmunology.

[146] Su H., Wang Y., Li H. (2021). RNA m6A methylation regulators multi-omics analysis in prostate cancer. Front. Genet..

[147] Meng J., Lu X., Jin C., Zhou Y., Ge Q., Zhang M., Zhou J., Hao Z., Yan F., Liang C. (2021). Integrated Multi-Omics Data Reveals the Molecular Subtypes of Prostate Cancer. bioRxiv.

[148] Liberini V., Laudicella R., Balma M., Nicolotti D.G., Buschiazzo A., Grimaldi S., Lorenzon L., Bianchi A., Peano S., Bartolotta T.V. (2022). Radiomics and artificial intelligence in prostate cancer: New tools for molecular hybrid imaging and theragnostics. Eur. Radiol. Exp..

[149] Qi Y., Zhao T., Han M. (2022). The application of radiomics in predicting gene mutations in cancer. Eur. Radiol..

[150] Prostate MRI and Ultrasound with Pathology and Coordinates of Tracked Biopsy (Prostate-MRI-US-Biopsy). https://wiki.cancerimagingarchive.net/pages/viewpage.action?pageId=68550661.

[151] The Cancer Imaging Archive. https://www.cancerimagingarchive.net/collections/.

[152] PESO: Prostate Epithelium Segmentation on H&E-Stained Prostatectomy Whole Slide Images. https://i2cvb.github.io/#prostate.

[153] Grand Challenge. https://grand-challenge.org/algorithms/bpmri-cspca-detection-report-guided-annotations/jobs/f26c1107-c2a4-4f21-93fe-91ce908ee365/.

[154] MICCAI Grand Challenge: Prostate MR Image Segmentation 2012. https://promise12.grand-challenge.org/.

[155] A Multi-site Dataset for Prostate MRI Segmentation. https://liuquande.github.io/SAML/.

[156] Annotated MRI and Ultrasound Volume Images of the Prostate. https://zenodo.org/record/16396#.ZDLEbHZByYq.

[157] The Cancer Imaging Archive. https://wiki.cancerimagingarchive.net/display/Public/Collections.

[158] SPIE-AAPM-NCI PROSTATEx Challenges (PROSTATEx). https://wiki.cancerimagingarchive.net/pages/viewpage.action?pageId=23691656.

[159] Prostate Tumor Warning Dataset. https://www.ncmi.cn/phda/dataDetails.do?id=CSTR:A0006.11.A0005.201905.000531-V1.0.

[160] Medical Segmentation Decathlon Generalisable 3D Semantic Segmentation. http://medicaldecathlon.com/.

[161] Prostate cANcer graDe Assessment (PANDA) Challenge Prostate Cancer Diagnosis Using the Gleason Grading System. https://www.kaggle.com/competitions/prostate-cancer-grade-assessment/data.

[162] Peng K., Mathur A., Narayanan A. (2021). Mitigating dataset harms requires stewardship: Lessons from 1000 papers. arXiv.

[163] (2016). Regulation (EU) 2016/679 of the European Parliament and of the Council. Off. J. Eur. Union.

[164] Chaddad A., Peng J., Xu J., Bouridane A. (2023). Survey of Explainable AI Techniques in Healthcare. Sensors.

[165] Reyes M., Meier R., Pereira S., Silva C.A., Dahlweid F.M., Tengg-Kobligk H.v., Summers R.M., Wiest R. (2020). On the interpretability of artificial intelligence in radiology: Challenges and opportunities. Radiol. Artif. Intell..

[166] Kraaijveld R.C., Philippens M.E., Eppinga W.S., Jurgenliemk-Schulz I.M., Gilhuijs K.G., Kroon P.S., van der Velden B.H. (2022). Multi-modal volumetric concept activation to explain detection and classification of metastatic prostate cancer on PSMA-PET/CT. Proceedings of the Interpretability of Machine Intelligence in Medical Image Computing: 5th International Workshop, iMIMIC 2022, Held in Conjunction with MICCAI 2022.

[167] Hassan M.R., Islam M.F., Uddin M.Z., Ghoshal G., Hassan M.M., Huda S., Fortino G. (2022). Prostate cancer classification from ultrasound and MRI images using deep learning based Explainable Artificial Intelligence. Future Gener. Comput. Syst..

[168] Gentile F., La Civita E., Della Ventura B., Ferro M., Cennamo M., Bruzzese D., Crocetto F., Velotta R., Terracciano D. (2022). A combinatorial neural network analysis reveals a synergistic behaviour of multiparametric magnetic resonance and prostate health index in the identification of clinically significant prostate cancer. Clin. Genitourin. Cancer.

[169] Rapisarda S., Bada M., Crocetto F., Barone B., Arcaniolo D., Polara A., Imbimbo C., Grosso G. (2020). The role of multiparametric resonance and biopsy in prostate cancer detection: Comparison with definitive histological report after laparoscopic/robotic radical prostatectomy. Abdom. Radiol..

[170] Wei J.T., Dunn R.L., Litwin M.S., Sandler H.M., Sanda M.G. (2000). Development and validation of the expanded prostate cancer index composite (EPIC) for comprehensive assessment of health-related quality of life in men with prostate cancer. Urology.

[171] Patel M., Turchan W.T., Morris C.G., Augustine D., Wu T., Oto A., Zagaja G.P., Liauw S.L. (2023). A contemporary report of low-dose-rate brachytherapy for prostate cancer using MRI for risk stratification: Disease outcomes and patient-reported quality of life. Cancers.

[172] Sanmamed N., Adleman J., Berlin A., Borg J., Lao B., Weersink R., Simeonov A., Rink A., Beiki-Ardakani A., Menard C. (2023). Acute toxicity and health-related quality of life outcomes of localized prostate cancer patients treated with magnetic resonance imaging-guided high-dose-rate brachytherapy: A prospective phase II trial. Brachytherapy.

[173] Reinikainen P., Lehtonen M., Lehtinen I., Luukkaala T., Sintonen H., Kellokumpu-Lehtinen P.L. (2023). Health-related quality of life of patients treated with different fractionation schedules for early prostate cancer compared to the age-standardized general male population. Clin. Genitourin. Cancer.

[174] Zhang X., Xia Q., Xu J. (2023). Palliative TURP Combined with Intermittent ADT Is A Curative Therapy to Some Elderly Men with Localized Prostate Adenocarcinoma. J. Cancer.

